# IL-8–driven neutrophil NETosis triggers endothelial apoptosis and exacerbates preeclampsia

**DOI:** 10.1186/s12967-026-08084-3

**Published:** 2026-04-04

**Authors:** Wushan Li, Xinyuan Li, Xuemei Liu, Mingjie Zhang, Wei Li, Xinlin Jiao, Fengchun Gao, Baoxia Cui

**Affiliations:** 1 Department of Obstetrics and Gynecology, Cheeloo College of Medicine, Qilu Hospital of Shandong University, Shandong University, 107 Cultural West Road, Jinan, Shandong Province 250012 China; 2 Department of Immunology, Shandong Provincial Key Laboratory of Infection & Immunology, School of Basic Medical Sciences, Cheeloo College of Medicine, Shandong University, Jinan, Shandong Province 250012 China; 3 Department of Obstetrics, Jinan Maternity and Child Care Hospital, Shandong First Medical University, Jinan, Shandong Province 250000 China

**Keywords:** Preeclampsia, IL-8/CXCL8, Neutrophil extracellular traps, Endothelial cell apoptosis

## Abstract

**Background:**

Preeclampsia (PE) is a major cause of maternal and perinatal morbidity and mortality worldwide, characterized by hypertension, proteinuria, and placental dysfunction. Increasing evidence implicates aberrant immune activation and vascular injury in PE pathogenesis, but the upstream signals and cellular mechanisms remain incompletely understood.

**Methods:**

We integrated transcriptomic profiling, maternal serum cytokine analysis, and placental immunohistochemistry to identify dysregulated chemokines. Human and murine trophoblasts were stimulated under hypoxia or LPS challenge to assess IL-8/CXCL1 production. Functional assays of NET formation and endothelial apoptosis were conducted in trophoblast–neutrophil–endothelium co-culture systems. Finally, we tested the therapeutic effects of neutralizing anti-CXCL1 antibody or the CXCR1/2 inhibitor SX682 in LPS-induced murine models of PE.

**Results:**

We found that IL-8 was markedly elevated in PE patients and correlated with disease severity. Hypoxia- or LPS-stimulated trophoblasts secreted abundant IL-8/CXCL1, which recruited and activated neutrophils to undergo NETosis. NETs directly induced endothelial mitochondrial dysfunction and apoptosis, resulting in vascular injury. Pharmacological blockade of IL-8 signaling, either by CXCL1 neutralization or CXCR1/2 inhibition, significantly ameliorated hypertension, proteinuria, and fetal growth restriction in murine PE models, without altering placental weight or gross morphology.

**Conclusion:**

Our findings define a trophoblast–neutrophil–endothelium axis in which IL-8–driven NETosis exacerbates vascular pathology in PE. Targeting IL-8/CXCL1-CXCR1/2 signaling interrupts this pathogenic circuit, restores endothelial function, and improves maternal and fetal outcomes. These results highlight IL-8 signaling as a promising therapeutic target for PE.

**Graphical Abstract:**

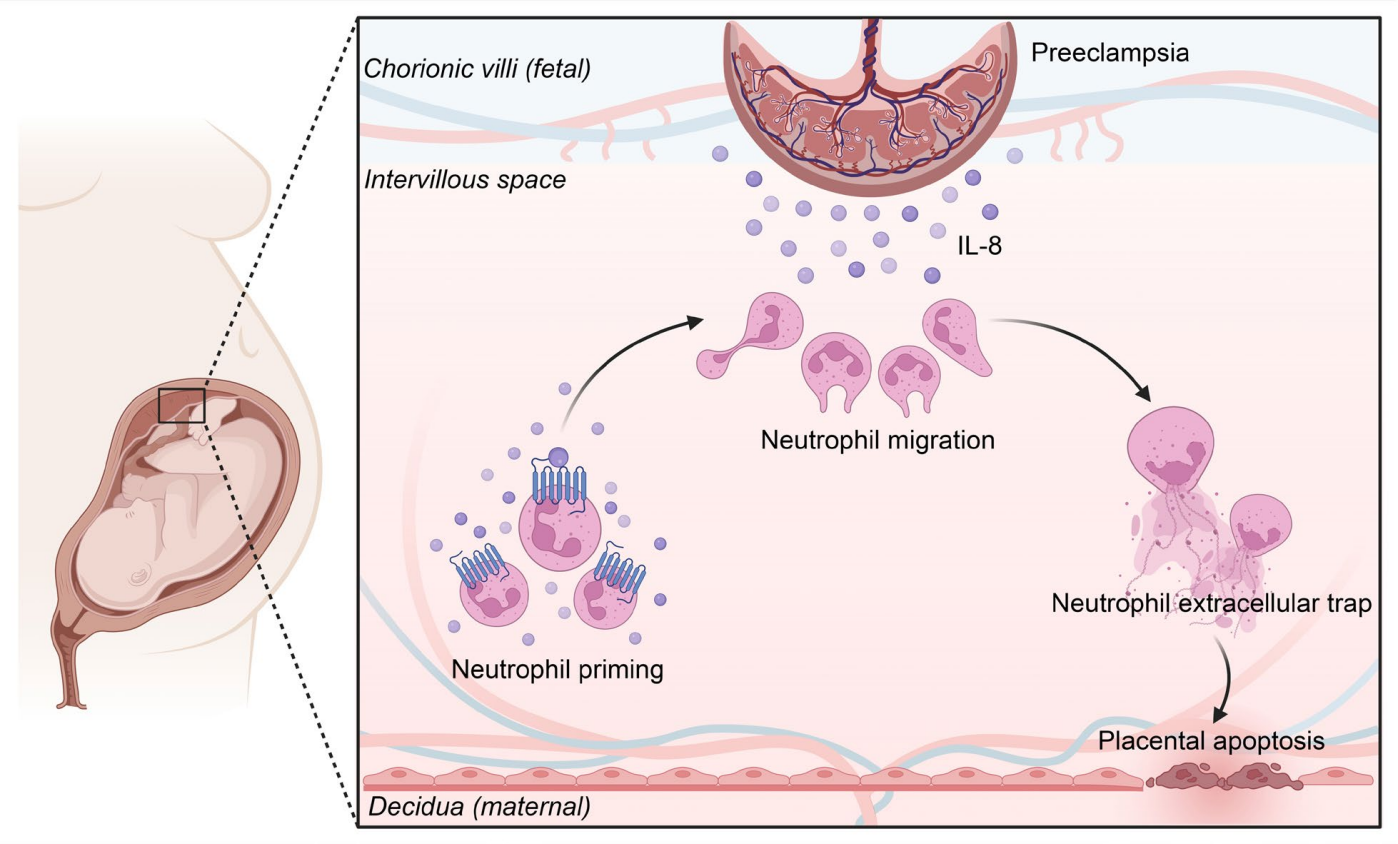

**Supplementary Information:**

The online version contains supplementary material available at 10.1186/s12967-026-08084-3.

## Introduction

Preeclampsia (PE) is a serious pregnancy-specific syndrome that affects 2%–8% of pregnancies worldwide and remains one of the leading causes of maternal and perinatal morbidity and mortality [1–2]. Clinically, PE is characterized by new-onset hypertension and proteinuria after 20 weeks of gestation, often accompanied by fetal growth restriction, placental insufficiency, and multi-organ dysfunction [3–4]. These manifestations reflect a systemic vascular disorder, in which widespread endothelial injury and dysregulated placental function play central roles [5–6]. Although advances in prenatal care have improved maternal and fetal outcomes, the only definitive treatment for PE remains delivery of the placenta, which may necessitate preterm birth and expose the neonate to significant complications [7]. This limitation underscores an urgent need to elucidate the molecular and cellular mechanisms underlying PE pathogenesis and to identify novel therapeutic targets.

Accumulating evidence implicates disrupted maternal immune responses as a key driver of PE [8–12]. In normal pregnancy, immune adaptations enable tolerance of the semi-allogeneic fetus while maintaining host defense [13]. In PE, this balance is perturbed, leading to excessive inflammatory activation and vascular injury [14–15]. Neutrophils, the most abundant leukocytes in peripheral blood, are increasingly recognized as critical mediators of this inflammatory pathology [16]. Beyond their traditional antimicrobial functions, neutrophils can contribute to sterile inflammation, particularly through the formation of neutrophil extracellular traps (NETs)—web-like structures composed of decondensed chromatin decorated with cytotoxic enzymes such as neutrophil elastase and myeloperoxidase [17–18]. NET formation, or NETosis, is an antimicrobial defense mechanism, yet when dysregulated it can cause collateral damage to host tissues, including the vascular endothelium [19–20]. Elevated circulating neutrophil counts have been reported in PE; however, the functional relevance of neutrophil activation, the upstream cues that drive NET formation, and the direct impact of NETs on maternal vascular pathology remain poorly understood [21].

The placenta is a major source of immune modulators during pregnancy and is highly sensitive to environmental stress. Hypoxia, a hallmark of placental malperfusion in PE, is a potent inducer of pro-inflammatory mediators [22–23]. In addition, systemic inflammation and microbial components, such as lipopolysaccharide (LPS), can further exacerbate placental immune activation [24]. Among the cytokines and chemokines induced under such conditions, interleukin-8 (IL-8, also known as CXCL8) stands out as a strong neutrophil chemoattractant and activator [25]. IL-8 exerts its biological effects primarily through binding to the G protein–coupled receptors CXCR1 and CXCR2, leading to neutrophil recruitment, degranulation, and respiratory burst, as well as priming of NET formation [26]. Elevated IL-8 has been documented in inflammatory and vascular diseases, including sepsis, acute lung injury, and atherosclerosis, where it is often associated with endothelial dysfunction. In pregnancy disorders, IL-8 upregulation has been observed in amniotic fluid, maternal serum, and placental tissue, but its mechanistic contribution to PE pathogenesis has not been fully elucidated.

Recent studies suggest that IL-8 not only recruits neutrophils to sites of inflammation but also potentiates their ability to undergo NETosis in sterile inflammatory environments. Given the close anatomical proximity of trophoblasts to maternal blood and their role as primary immune modulators at the maternal–fetal interface, it is plausible that trophoblast-derived IL-8 could be a critical upstream signal linking placental stress to systemic vascular injury in PE. However, direct mechanistic evidence connecting trophoblast IL-8 production, neutrophil NETosis, and endothelial cell damage in PE is lacking. Furthermore, the therapeutic potential of targeting the IL-8–CXCR1/2 axis in PE has not been explored in vivo.

Here, we investigated the role of trophoblast-derived IL-8 in driving neutrophil NETosis and endothelial apoptosis in the pathogenesis of PE. By integrating transcriptomic profiling, serum cytokine analysis, and placental immunohistochemistry from PE patients, we demonstrate that IL-8 is markedly elevated in PE and correlates with disease severity. In vitro, we show that hypoxia or LPS stimulation induces robust IL-8 secretion by trophoblasts, which recruits and activates neutrophils to form NETs. These NETs directly induce apoptosis in vascular endothelial cells, providing a mechanistic link between placental stress and systemic endothelial dysfunction. Using a murine model of PE, we further demonstrate that blockade of IL-8 signaling via CXCL8-neutralizing antibody or pharmacological inhibition of CXCR1/2 significantly reduces maternal hypertension, proteinuria, and fetal growth restriction. Together, these findings identify a trophoblast–neutrophil–endothelium axis in which IL-8–driven NETosis exacerbates PE, and establish IL-8 signaling as a promising therapeutic target for mitigating maternal and fetal complications.

## Materials and methods

### Cell culture and neutrophil isolation

Human umbilical vein endothelial cells (HUVECs; Chinese Academy of Sciences Cell Bank, Cat. No. GNHu23, RRID: CVCL2959) and human trophoblast cells (HTR-8/SVneo; Chinese Academy of Sciences Cell Bank, Cat. No. SCSP-5203, RRID: CVCL7162) were purchased on April 18, 2025. HUVECs were maintained in vascular cell basal medium supplemented with endothelial cell growth factors, while HTR-8/SVneo cells were cultured in RPMI-1640 medium with 10% fetal bovine serum (FBS; Gibco, RRID: SCR_013923), 100 µg/mL penicillin, and 100 U/mL streptomycin. All cell lines were authenticated by short tandem repeat (STR) profiling and tested negative for mycoplasma contamination prior to experiments.

Murine neutrophils were isolated from bone marrow using magnetic-activated cell sorting (MACS) with anti-Ly6G microbeads (Miltenyi Biotec, Cat. No. 130-120-337, RRID: AB_2784239), while human neutrophils were purified from peripheral blood using anti-CD16 microbeads (Miltenyi Biotec, Cat. No. 130-045-701, RRID: AB_2665454), according to the manufacturer’s instructions. Isolated neutrophils were maintained in RPMI-1640 medium (Gibco, RRID: SCR_013916) containing 10% FBS. All cells were cultured at 37 °C in a humidified incubator with 5% CO₂.

### Co-culture system and stimulation

To model trophoblast-neutrophil-endothelial interactions, trophoblasts were exposed to hypoxia (1% O₂) or lipopolysaccharide (LPS; 100 ng/mL, Sigma-Aldrich), and the cell supernatant was collected. Endothelial cells (1 × 10⁵) were seeded into 12-well plates and allowed to adhere overnight, then endothelial cells were cocultured with neutrophils and trophoblast supernatant with/without neutralizing antibody against CXCL8/CXCL1 (2 µg/mL) or the CXCR1/2 inhibitor SX682 (Selleck, 5 µM, RRID: SCR_021157). These conditions were applied for all assays described below unless otherwise specified.

### Immunofluorescence staining of placental tissues

Placental tissues from normotensive pregnancies and patients with preeclampsia were collected immediately after delivery and fixed in 4% paraformaldehyde. After paraffin embedding, tissue sections (5 μm) were prepared for immunofluorescence staining. Sections were deparaffinized, rehydrated, and subjected to antigen retrieval in citrate buffer (pH 6.0). After blocking with 5% bovine serum albumin for 1 h at room temperature, sections were incubated overnight at 4 °C with primary antibodies against IL-8 (Abcam, Cat: ab282000) and cytokeratin-7 (CK7; Abcam, Cat: ab9021). After washing with PBS, sections were incubated with species-appropriate fluorescent secondary antibodies for 1 h at room temperature in the dark. Nuclei were counterstained with DAPI, and slides were mounted with antifade medium. Images were acquired using a confocal fluorescence microscope and processed with ImageJ software.

### Isolation of neutrophil extracellular traps (NETs)

NETs were isolated following induction of NETosis. Briefly, freshly isolated human or mouse neutrophils were resuspended at 1 × 10⁶ cells/mL and seeded onto poly-L-lysine–coated plates to facilitate adhesion. After 30 min incubation at 37 °C with 5% CO₂, NETosis was induced by stimulation with recombinant human IL-8 or murine CXCL1 for 6 h. NET formation was verified by fluorescence detection of extracellular DNA structures. Culture medium was then removed, wells were gently washed with PBS to eliminate non-adherent cells, and NETs structures were detached by gentle pipetting in PBS. The suspension was sequentially centrifuged (500 × g for 5 min followed by 2,000 × g for 10 min) to remove intact cells and debris, and the resulting supernatant was collected as the NET-rich fraction. Extracellular DNA content was quantified using the PicoGreen dsDNA assay. In DNase control experiments, NET preparations were treated with DNase I prior to endothelial cell exposure to degrade extracellular DNA structures.

### Measurement of ROS

Intracellular ROS levels in endothelial cells were measured using the DCFH-DA assay (Beyotime, S0033S; Ex = 488 nm, Em = 525 nm). After 24 h co-culture, endothelial cells were collected, incubated with 10 µM DCFH-DA at 37 °C for 30 min, washed twice with PBS, and analyzed by flow cytometry. A total of 10⁴ events per sample were recorded, and data were analyzed using FlowJo v10.

### Intracellular calcium measurement

Cytosolic Ca²⁺ levels were quantified using the Fluo-4 AM kit (Beyotime, S1060; Ex = 488 nm, Em = 512–520 nm). Endothelial cells were incubated with 2 µM Fluo-4 AM for 30 min at 37 °C in the dark, washed twice with PBS, and analyzed by flow cytometry as above.

### TUNEL assay

Apoptotic endothelial cells were detected using a TUNEL red fluorescence kit (Beyotime, C1090; Ex = 550 nm, Em = 570 nm). After 24 h co-culture, cells were incubated with 50 µL TUNEL reaction mixture for 1 h at 37 °C in the dark and analyzed by flow cytometry.

### Cell apoptosis detection

For Annexin V/propidium iodide (PI) staining, cells were labeled with FITC-Annexin V and PI (BD Biosciences, 556547) for 10 min at room temperature in the dark and analyzed immediately by flow cytometry (Ex = 488 nm, Em = 525 nm for FITC; Em = 585 nm for PI).

### Live/dead cell staining

Endothelial cell viability was evaluated by Calcein-AM/PI double staining (Yeasen, 40747ES76). After 48 h co-culture, cells were incubated with 2 µM Calcein-AM and 4.5 µM PI for 15 min at 37 °C in the dark. Images were acquired using an Andor Dragonfly 200 confocal system and analyzed with Fusion software.

### NETs visualization

Neutrophil extracellular traps (NETs) were visualized using SYTOX™ Green (Invitrogen, S7020; 500 nM). After 48 h co-culture, neutrophils were fixed with 4% paraformaldehyde for 15 min, washed twice with PBS, incubated with SYTOX Green for 30 min at room temperature in the dark, and imaged using the Andor Dragonfly 200 system.

### Western blotting

Endothelial cells were lysed in RIPA buffer containing protease and phosphatase inhibitors, and protein concentrations were determined by BCA assay. Equal amounts of protein were separated by SDS-PAGE, transferred to PVDF membranes, and blocked in 5% nonfat milk. Membranes were incubated overnight at 4 °C with primary antibodies against p38 (Cell Signaling Technology, #9212, RRID: AB_330713), phospho-p38 (Cell Signaling Technology, #4511, RRID: AB_2139682), BCL2 (Cell Signaling Technology, #3498, RRID: AB_10692504), ERK (Servicebio, GB11560-100, RRID: AB_2921163), phospho-ERK (Servicebio, GB11004-100, RRID: AB_2921164), cleaved caspase-3 (Cell Signaling Technology, #9664, RRID: AB_2070042), and β-actin (Cell Signaling Technology, #4967, RRID: AB_330288), followed by HRP-conjugated secondary antibodies. Signals were detected using ECL reagents and imaged with a chemiluminescence system.

### Animal models of preeclampsia

All animal procedures were approved by the Institutional Animal Care and Use Committee. Female C57BL/6 mice (8–10 weeks) were mated at a 2:1 ratio, and vaginal plugs detected the next morning were designated gestational day 0.5 (GD0.5). *LPS-induced inflammatory PE model*: Pregnant mice were randomized to control or PE groups. From GD7.5 to GD17.5, PE mice received daily intraperitoneal injections of LPS (1 µg/kg in 100 µL PBS), whereas controls received PBS. In therapeutic experiments, SX682 (50 mg/kg, oral gavage, twice daily) or anti-CXCL8/CXCL1 antibody (10 mg/kg, intraperitoneal, every other day) was administered from GD7.5 to GD17.5. *Adv-sFlt-1 overexpression model*: Recombinant adenovirus carrying sFlt-1 (Adv–sFlt-1; 1 × 10⁹ PFU in 100 µL PBS) was injected via the tail vein from GD7.5 to GD17.5, control group was received Adv-GFP. Serum sFlt-1 levels were measured on GD14 by ELISA. All animal procedures were conducted under protocols approved by the Medical Ethics Committee of Jinan Maternal and Child Health Hospital (KYR-25-003) and in accordance with National Institutes of Health standards.

### Physiological assessments

Systolic blood pressure was measured by tail-cuff plethysmography. Urine was collected over 24 h in metabolic cages, and protein concentration was quantified by BCA assay. On GD18.5, fetuses and placentas were weighed, and tissues were collected for histological and flow cytometric analyses.

### Statistics

Data are presented as mean ± standard error of the mean (SEM). Statistical comparisons between two groups were performed using unpaired two-tailed Student’s t-tests. For multiple group comparisons, one-way ANOVA followed by Tukey’s post hoc test was used. *P* < 0.05 was considered statistically significant. All statistical analyses were performed using GraphPad Prism 9.

## Results

### Elevated trophoblast-derived IL-8/CXCL1 correlates with neutrophil infiltration and disease severity in human and murine preeclampsia

**Fig. 1:**
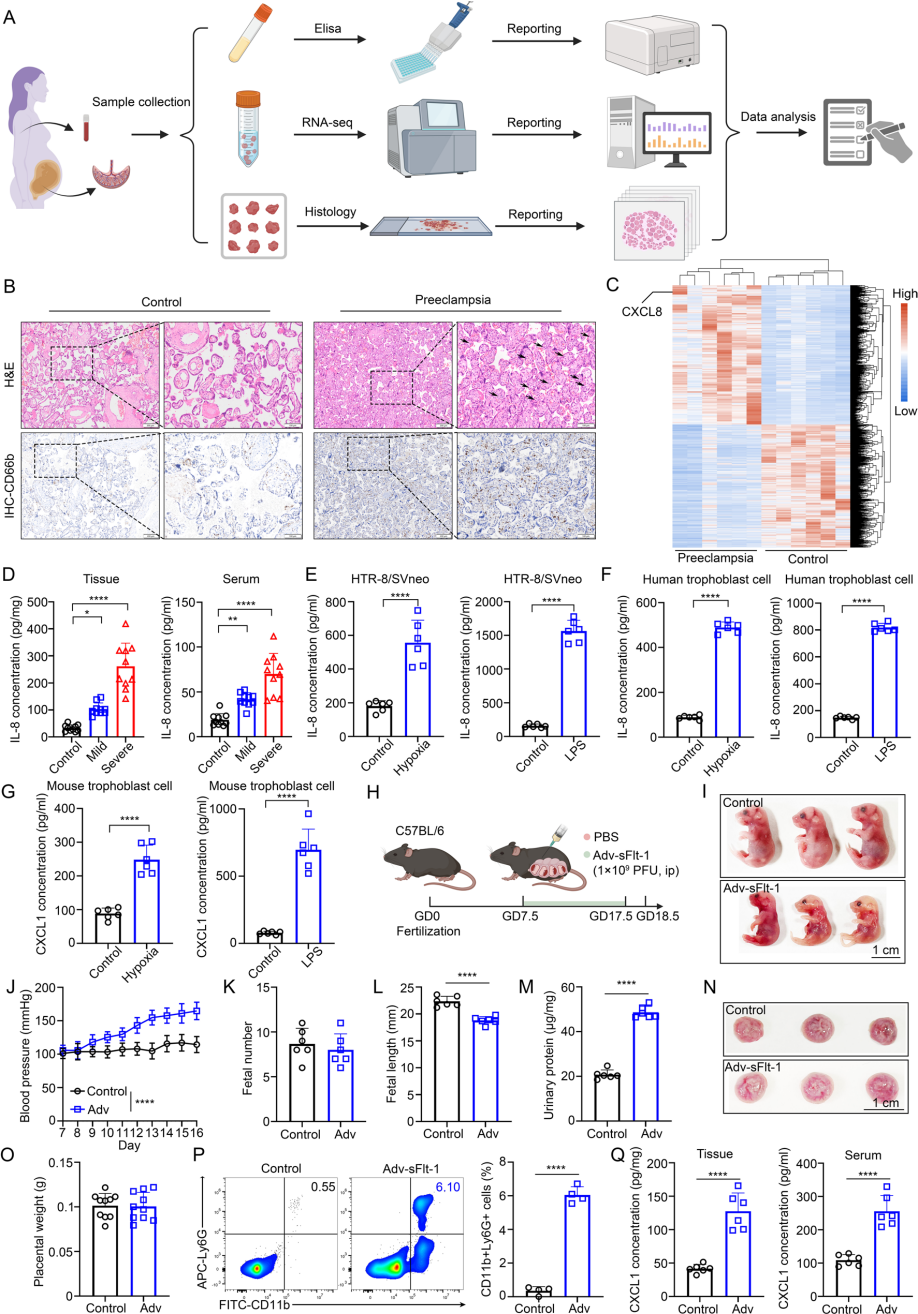
Elevated trophoblast-derived IL-8/CXCL1 correlates with neutrophil infiltration and disease severity in preeclampsia patients and mouse models. (**A**) Experimental design and methodology for analysis of human samples. (**B**) H&E and IHC (CD66b for neutrophils) staining of placental tissue from healthy donor and preeclampsia patient. Black arrows indicate leukocyte infiltration. Scale bar, 100 μm and 200 μm. (**C**) Differential expression profile between healthy donor clusters and preeclampsia patient (*n* = 6 biologically independent samples per group). (**D**) IL-8 concentration in tissue and serum from healthy donor and preeclampsia patient (mild and severe) (*n* = 10). (**E**) IL-8 concentration in supernatant of HTR-8/SVneo cells treated with hypoxia and LPS (*n* = 6). (**F**) IL-8 concentration in supernatant of human trophoblast cells treated with hypoxia and LPS (*n* = 6). (**G**) CXCL1 concentration in supernatant of mouse trophoblast cells treated with hypoxia and LPS (*n* = 6). (**H**) Schematic of in vivo experiment of C57BL/6 mice treated with Adv-sFlt-1. (**I**) Representative images of fetal mice treated with Adv-sFlt-1 (*n* = 3 dams per group). Scale bar, 1 cm. (**J**) Statistical analysis of blood pressure (*n* = 3 dams per group). (**K**) Statistical analysis of fetal number (*n* = 6 litters per group). (**L**) Statistical analysis of fetal length (*n* = 6 litters per group). (**M**) Statistical analysis of urinary protein concentration in pregnant mice (*n* = 6 litters per group). (**N**) Representative images of placenta from mice treated with Adv-sFlt-1 (*n* = 3 dams per group). Scale bar, 1 cm. (**O**) Statistical analysis of placental weight (*n* = 10). (**P**) Gating strategy, representative plots and quantification diagrams of mouse CD11b^+^Ly6G^+^ neutrophils in placental tissue from mice treated with Adv-sFlt-1. (**Q**) CXCL1 concentration in tissue and serum from mice treated with Adv-sFlt-1 (*n* = 6). *p < 0.05, **p < 0.01, ****p < 0.0001. Statistical values were calculated using one-way ANOVA (**D**), Student’s t-test (**E**-**G**, **I**, **K**, **L**, **M**, **P**, **Q**), two-way ANOVA (**J**)

To elucidate the inflammatory landscape of PE, we collected placental tissue and peripheral blood samples from normotensive pregnant women and patients diagnosed with either mild or severe PE (Fig. 1A and Supplementary Table S1). Histological examination revealed pronounced leukocyte infiltration in PE placentas, accompanied by substantial accumulation of CD66b⁺ neutrophils, as shown by immunohistochemistry (Fig. 1B). Bulk RNA-seq analysis of placental tissues further demonstrated a distinct transcriptional profile in PE, with IL-8 (CXCL8) significantly upregulated (Fig. 1C). Quantification by ELISA confirmed that IL-8 levels were significantly elevated in both placental tissue and maternal serum, correlating positively with PE severity (Fig. 1D). These data support a strong association between IL-8 upregulation and neutrophil infiltration in PE.

To investigate the cellular source of IL-8, we stimulated the human trophoblast cell line HTR-8/SVneo and human primary trophoblast cells with either hypoxia or lipopolysaccharide (LPS) —two pathological cues relevant to PE. Both conditions significantly increased IL-8 secretion into the supernatant (Fig. 1E-F). Next, to determine whether trophoblasts are the primary source of elevated IL-8 in PE placentas, we performed immunofluorescence co-staining on placental tissue sections from healthy controls and PE patients. Sections were co-stained with antibodies against IL-8 and cytokeratin 7, a specific marker of trophoblast cells. As shown in Supplementary Figure S1A, PE placental tissues showed intense IL-8 signal that extensively co-localized with CK7 compared with healthy controls. These results provide direct evidence that trophoblasts are a major cellular source of the elevated IL-8 specifically in preeclamptic placentas. Similarly, primary trophoblast cells isolated from pregnant mice exhibited elevated release of CXCL1, the murine functional homolog of IL-8, in response to the same stimuli, suggesting trophoblast cell-derived chemokine signaling as a conserved response across species (Fig. 1G).

To establish an in vivo model, we injected pregnant C57BL/6 mice with an adenoviral vector encoding soluble fms-like tyrosine kinase-1 (Adv-sFlt-1) at gestational day 7.5 (GD7.5) to mimic PE-like conditions (Fig. 1H). Compared with controls, the Adv-sFlt-1-treated group exhibited decreased body size of fetal mice, elevated systolic blood pressure, increased urinary protein excretion, and reduced fetal length, while fetal numbers and placental weights remained unchanged (Fig. 1I-O). These findings phenocopy key clinical features of human PE. Flow cytometry analysis of placental single-cell suspensions revealed significantly increased infiltration of CD11b⁺Ly6G⁺ neutrophils in Adv-sFlt-1-treated mice, accompanied by elevated CXCL1 levels in both placental tissue and maternal serum (Fig. 1P-Q).

To validate these findings in an alternative model, we established an LPS-induced PE-like model in pregnant mice (Supplementary Figure S1B). Consistent with the Adv-sFlt-1 model, LPS exposure resulted in reduced fetal growth, increased maternal blood pressure, elevated proteinuria, and unaltered fetal numbers and placental weights (Supplementary Figure S1C-S1I). Flow cytometry again confirmed enhanced neutrophil infiltration, and ELISA revealed increased CXCL1 concentrations in both placental and serum samples (Supplementary Figure S1J-S1K).

Collectively, these data from human tissues, in vitro trophoblast cell models, and two independent mouse models converge to indicate that trophoblast-derived IL-8/CXCL1 is a key mediator of neutrophil infiltration in preeclampsia and correlates strongly with disease severity and vascular pathology.

### Hypoxia or LPS-stimulated trophoblasts promote IL-8–dependent neutrophil NETosis that triggers endothelial apoptosis and dysfunction

**Fig. 2:**
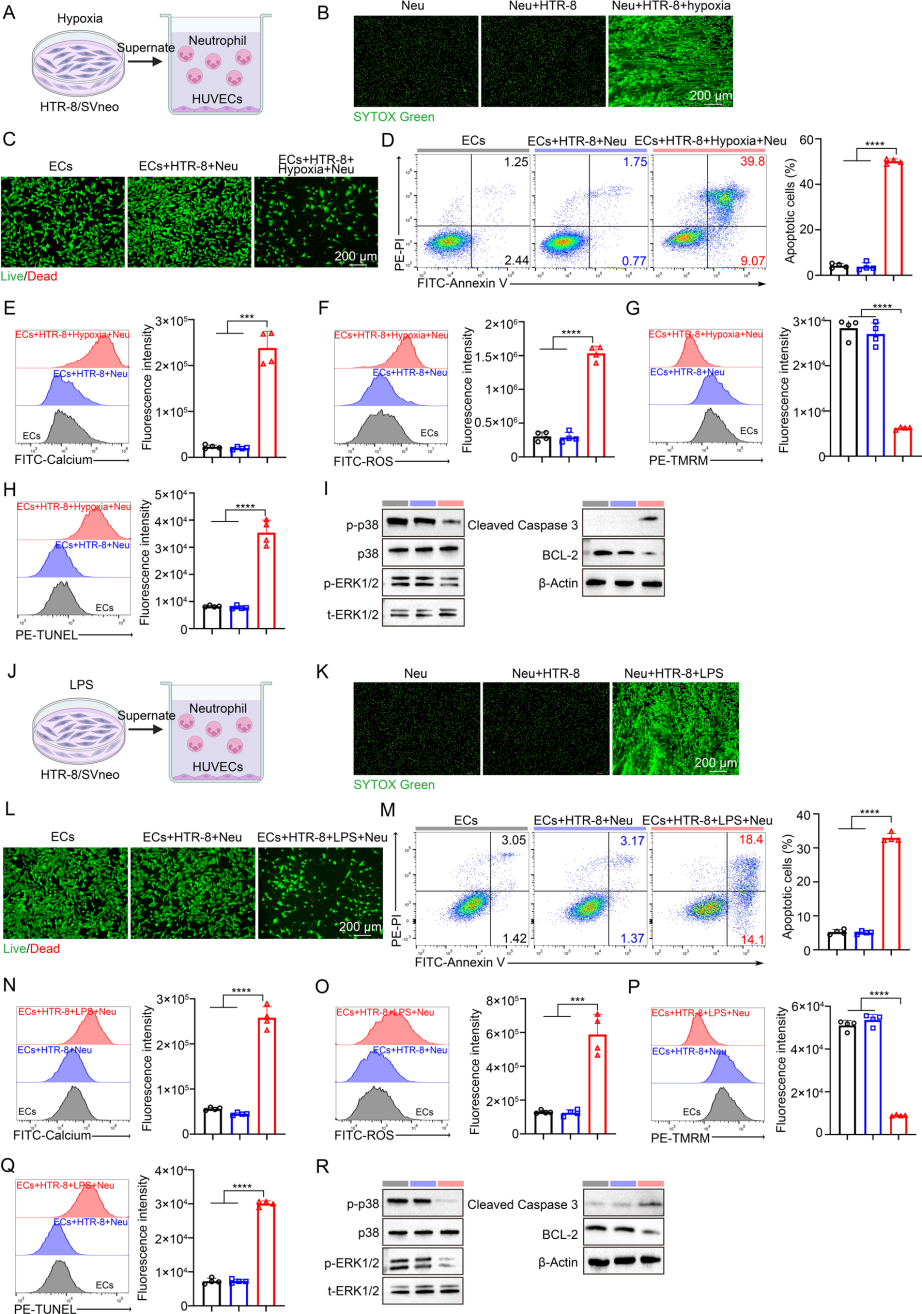
Hypoxia- or LPS-stimulated trophoblasts promote IL-8- dependent NETosis that triggers endothelial apoptosis and dysfunction. (**A**) Schematic illustration showing that HTR-8/SVneo cells were cultured under hypoxic conditions, and the collected supernatant was co-cultured with neutrophils and HUVECs. (**B**) Confocal microscopy images showing NET formation of neutrophils with different treatment. Scale bar: 200 μm. (**C**) Fluorescence images in live/dead staining experiments of HUVECs. Scale bar: 200 μm. (**D**) Representative flow cytometry plots and quantification of cell apoptosis of HUVECs (*n* = 4). (**E**) Representative plots and quantification diagrams showing intracellular calcium levels of HUVECs after treatment (*n* = 4). (**F**) Representative plots and quantification diagrams showing intracellular ROS levels of HUVECs after treatment (*n* = 4). (**G**) Representative plots and quantification diagrams showing TMRM levels of HUVECs after treatment (*n* = 4). (**H**) Representative plots and quantification diagrams showing TUNEL levels of HUVECs after treatment (*n* = 4). (**I**) Expression levels of p38, ERK1/2, caspase-3 and Bcl-2 from HUVECs with/without hypoxic treatment. (**J**) Schematic illustration showing that HTR-8/SVneo cells were cultured under LPS conditions, and the collected supernatant was co-cultured with neutrophils and HUVECs. (**K**) Confocal microscopy images showing NET formation of neutrophils with different treatment. Scale bar: 200 μm. (**L**) Fluorescence images in live/dead staining experiments of HUVECs. Scale bar: 200 μm. (**M**) Representative flow cytometry plots and quantification of cell apoptosis of HUVECs (*n* = 4). (**N**) Representative plots and quantification diagrams showing intracellular calcium levels of HUVECs after treatment (*n* = 4). (**O**) Representative plots and quantification diagrams showing intracellular ROS level of HUVECs after treatment (*n* = 4). (**P**) Representative plots and quantification diagrams showing TMRM level of HUVECs after treatment (*n* = 4). (**Q**) Representative plots and quantification diagrams showing TUNEL level of HUVECs after treatment (*n* = 4). (**R**) Expression and phosphorylation levels of p38, ERK1/2, caspase-3 and Bcl-2 from HUVECs with/without LPS treatment. ***p < 0.001, ****p < 0.0001. Statistical values were calculated using one-way ANOVA (**D**-**H**, **M**-**Q**)

To test whether trophoblast activation is sufficient to provoke a NETosis-endothelium injury cascade consistent with the IL-8/CXCL1 axis identified in Fig. 1, we exposed HTR-8/SVneo cells to hypoxia and applied the conditioned medium to neutrophils and HUVECs (Fig. 2A). Hypoxia-conditioned supernatants induced robust NET formation by neutrophils and reduced endothelial viability (Fig. 2B-C). Flow cytometry confirmed a significant increase in HUVEC apoptosis, accompanied by intracellular Ca²⁺ overload and ROS accumulation, loss of mitochondrial membrane potential (TMRM), and increased TUNEL signal (Fig. 2D-H). To ensure that the measured apoptotic signals were derived exclusively from endothelial cells and not from adherent neutrophils in the co-culture system, we employed an endothelial cell-specific gating strategy using CD144 (VE-cadherin) in flow cytometric analysis. As shown in Supplemental Figure S2A-S2E, neutrophils were CD144⁻ and could be clearly distinguished from CD144⁺ endothelial cells. The results obtained from CD144-gated endothelial cells were consistent with our original findings. Immunoblotting revealed coordinated alterations in the p38/ERK pathway together with elevated cleaved caspase-3 and reduced Bcl-2, consistent with activation of an apoptotic program downstream of NETosis (Fig. 2I).

This hypoxia-driven sequence was independently reproduced in a mouse setting, where conditioned media from hypoxia-treated primary trophoblasts promoted NETosis in neutrophils and apoptosis in MUVECs with concordant changes in Ca²⁺, ROS, TMRM and TUNEL and matching MAPK/caspase/Bcl-2 profiles (Supplementary Figure S3A-S3I). In a parallel paradigm, we stimulated HTR-8/SVneo cells with LPS and performed the same two-step co-culture (Fig. 2J). LPS-conditioned supernatants similarly triggered neutrophil NET formation and diminished HUVEC viability, with increased Annexin V/PI–defined apoptosis and the same constellation of biochemical stress and death readouts—elevated intracellular Ca²⁺ and ROS, loss of TMRM, and TUNEL positivity (Fig. 2K-Q). Immunoblot analyses again showed coordinated modulation of the p38/ERK axis, increased cleaved caspase-3 and decreased Bcl-2 under LPS-conditioned challenge (Fig. 2R). These effects were recapitulated in the mouse trophoblast-neutrophil-MUVEC system under LPS stimulation, confirming NETosis-linked endothelial injury with identical molecular signatures (Supplementary Figure S4A-S4I).

To further validate the cellular source of IL-8 and its role in NET-mediated endothelial injury, we isolated primary human trophoblasts from normal term placentas and stimulated them under hypoxic conditions or LPS. As shown in Supplemental Figure S5A-S5D, conditioned medium from hypoxia- or LPS-stimulated primary trophoblasts significantly induced NET formation and exhibited markedly increased HUVEC apoptosis. These results demonstrate that primary human trophoblasts, when exposed to PE-relevant stressors, secrete factors that promote neutrophil NETosis and subsequent endothelial injury, consistent with our findings using HTR-8/SVneo cells.

To determine whether direct cell-cell contact is required for neutrophil-mediated endothelial injury, we established a transwell co-culture system in which neutrophils were seeded in the upper chamber and HUVECs in the lower chamber. As shown in Supplemental Figure S5E-S5H, transwell co-culture with conditioned medium significantly increased HUVEC apoptosis under both hypoxia and LPS conditions, indicating that soluble factors are sufficient to mediate endothelial injury without requiring direct contact. To directly confirm the cytotoxic role of NETs, we added DNase I to the co-culture system to degrade NET structures. DNase I treatment significantly reduced HUVEC apoptosis in both hypoxia- and LPS-stimulated conditions, returning apoptosis levels toward baseline (Supplemental Figure S5E-S5H). These results demonstrate that NETs are critical mediators of trophoblast-induced endothelial cell death.

### IL-8 induces neutrophil NETosis to provoke mitochondrial dysfunction and apoptosis in endothelial cells

**Fig. 3:**
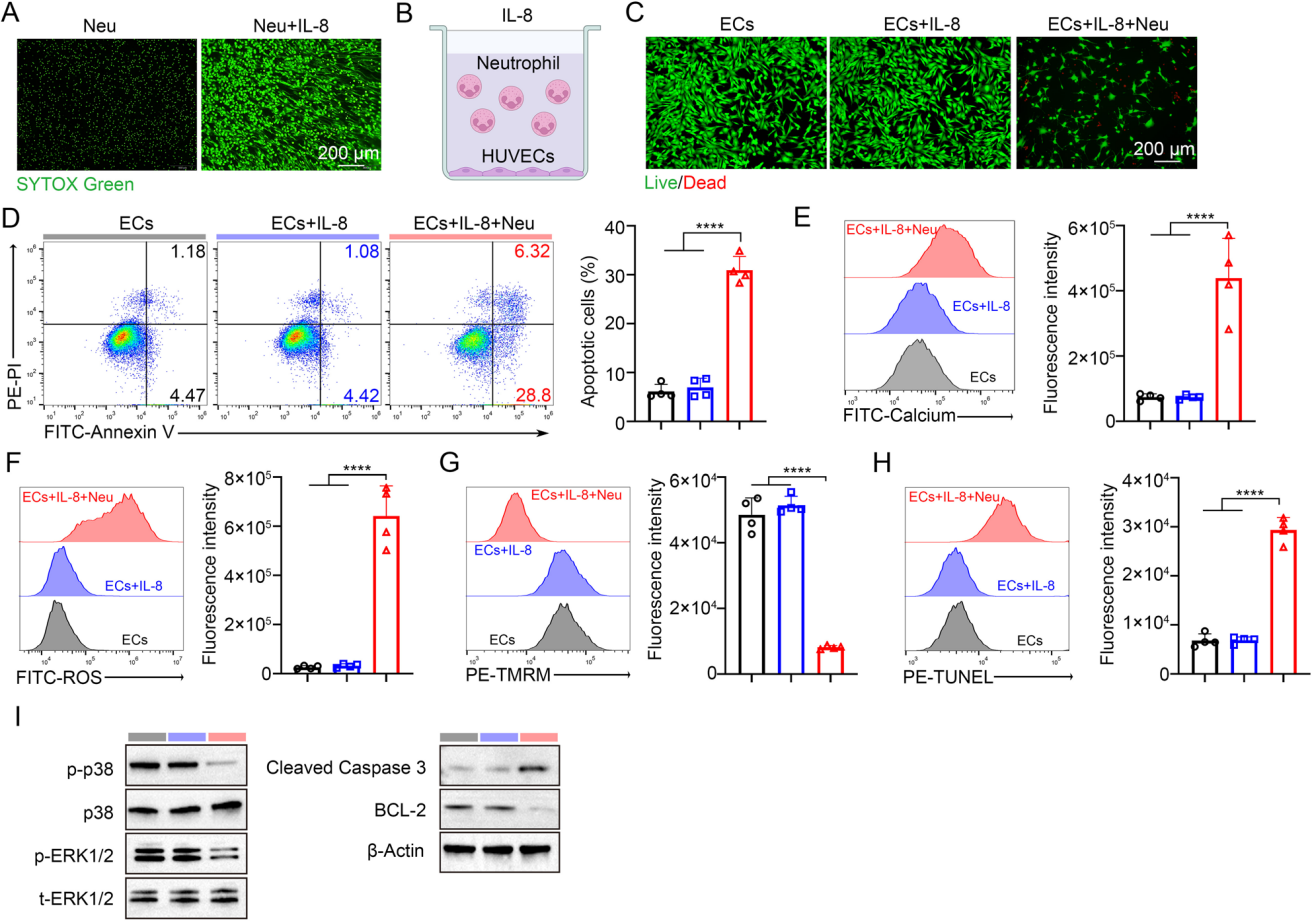
IL-8 induces neutrophil NETosis and promotes endothelial apoptosis through mitochondrial signaling pathways. (**A**) Confocal microscopy images showing NET formation of neutrophils with/without IL-8 treatment. Scale bar: 200 μm. (**B**) Schematic illustration showing that HUVECs were co-cultured with neutrophils under IL-8 conditions. (**C**) Fluorescence images in live/dead staining experiments of HUVECs. Scale bar: 200 μm. (**D**) Representative flow cytometry plots and quantification of cell apoptosis of HUVECs (*n* = 4). (**E**) Representative plots and quantification showing intracellular calcium levels of HUVECs after treatment (*n* = 4). (**F**) Representative plots and quantification showing intracellular ROS levels of HUVECs after treatment (*n* = 4). (**G**) Representative plots and quantification showing TMRM levels of HUVECs after treatment (*n* = 4). (**H**) Representative plots and quantification showing TUNEL levels of HUVECs after treatment (*n* = 4). (**I**) Expression and phosphorylation levels of p38, ERK1/2, caspase-3 and Bcl-2 from HUVECs with different treatment. ****p < 0.0001. Statistical values were calculated using one-way ANOVA (**D**-**H**)

Guided by the trophoblast-neutrophil-endothelium cascade defined above, we next asked whether IL-8 itself is sufficient to drive NETosis and consequent endothelial injury, and whether the downstream damage converges on mitochondrial stress signalling. Recombinant human IL-8 robustly induced NET release from purified human neutrophils, as visualized by SYTOX Green-based confocal imaging (Fig. 3A). We then established a reductionist co-culture in which HUVECs were exposed to neutrophils in the presence of IL-8 (Fig. 3B). Live/dead imaging revealed marked endothelial apoptosis in the IL-8-neutrophil condition, while IL-8 alone caused little overt toxicity (Fig. 3C). Quantitatively, Annexin V/PI flow cytometry demonstrated a significant increase in HUVEC apoptosis upon IL-8- driven neutrophil activation, indicating that IL-8-elicited NETosis is sufficient to compromise endothelial integrity (Fig. 3D).

We next profiled canonical hallmarks of mitochondrial distress that typify NET-mediated cytotoxicity. Relative to HUVECs alone or HUVECs exposed to IL-8 without neutrophils, co-culture with IL-8–stimulated neutrophils elicited intracellular Ca²⁺ overload, enhanced ROS production, and collapse of mitochondrial membrane potential, together with increased DNA fragmentation (Fig. 3E-H). Immunoblotting of HUVEC lysates after IL-8-neutrophil challenge showed coordinated remodeling of stress-death pathways: phosphorylation of p38 and ERK1/2 was diminished without changes in total protein levels, Bcl-2 was reduced, and cleaved caspase-3 was increased (Fig. 3I). These data illustrated mitochondrial dysfunction upstream of caspase activation and link NET-associated stress to a switch away from pro-survival MAPK signaling .

To test conservation across species and the generalizability of IL-8 family chemokines, we reproduced the paradigm with murine reagents using CXCL1. Exogenous CXCL1 was sufficient to trigger NET formation by mouse neutrophils and to provoke endothelial apoptosis in a MUVEC-neutrophil co-culture (Supplementary Figure S6A-S6C). As in the human system, CXCL1-driven neutrophil activation significantly increased MUVEC apoptosis, accompanied by Ca²⁺ influx, ROS accumulation, loss of TMRM signal and elevated TUNEL staining (Supplementary Figure S6D-S6H). Immunoblot analyses mirrored the human findings, with reduced p-p38 and p-ERK1/2, increased cleaved caspase-3, and decreased Bcl-2, while total p38 and ERK1/2 remained unchanged (Supplementary Figure S6I). To further confirm that NETs are the direct mediators of endothelial injury, we collected NETs derived from human neutrophils stimulated with IL-8 or mouse neutrophils stimulated with CXCL1, and applied to HUVEC or MUVEC cultures, respectively. As shown in Supplementary Figure S6J-S6L, NETs from IL-8/CXCL1-stimulated neutrophils significantly increased endothelial cell apoptosis compared to controls, and this effect was nearly abolished by DNase I treatment. Neutrophils alone had no direct cytotoxic effect on endothelial cells. These results confirm that NETs are the primary mediators of endothelial apoptosis in both human and murine systems.

Together with the upstream evidence that hypoxia- or LPS-activated trophoblasts secrete IL-8/CXCL1 and drive NETosis-dependent endothelial dysfunction, these results define a causal axis in which IL-8 family chemokines are both necessary in the trophoblast context and sufficient on their own to trigger NET-mediated mitochondrial stress culminating in endothelial apoptosis. This places IL-8/CXCL1 at the core of a trophoblast-neutrophil-endothelium triad and suggests that interrupting chemokine sensing or NET-mitochondria crosstalk could blunt endothelial damage at the maternal-fetal interface.

### CXCR1/2 blockade with SX682 curbs trophoblast-induced neutrophil NETosis and rescues endothelial function

**Fig. 4:**
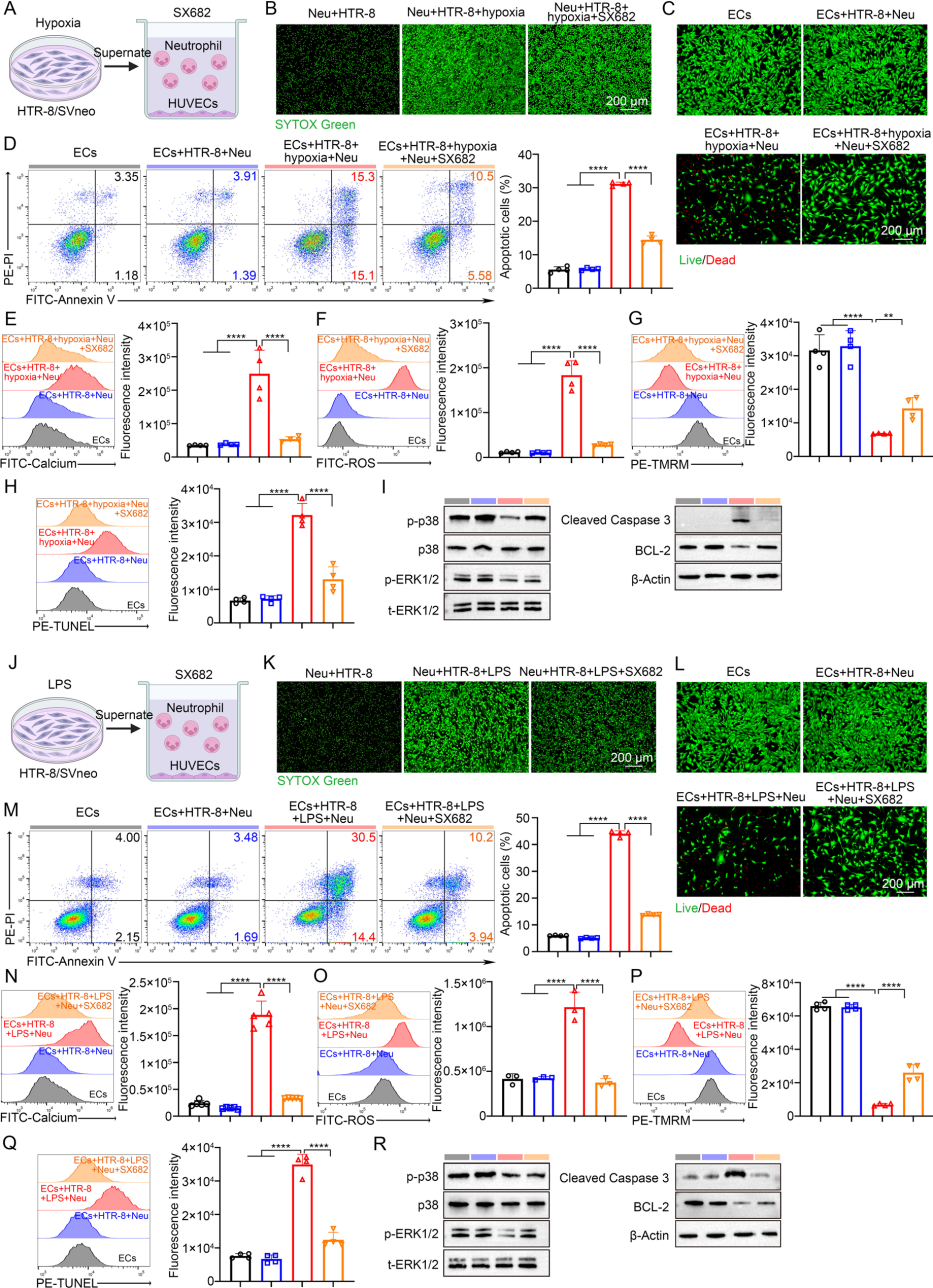
Inhibition of CXCR1/2 with SX682 suppresses trophoblast-induced neutrophil NETosis and protects endothelial cells from apoptosis and dysfunction. (**A**) Schematic illustration showing that HTR-8/SVneo cells were cultured under hypoxic conditions, and the collected supernatant was co-cultured with neutrophils and HUVECs under SX682 treatment. (**B**) Confocal microscopy images showing NET formation of neutrophils with different treatment. Scale bar: 200 μm. (**C**) Fluorescence images in live/dead staining experiments of HUVECs. Scale bar: 200 μm. (**D**) Representative flow cytometry plots and quantification of cell apoptosis of HUVECs (*n* = 4). (**E**) Representative plots and quantification showing intracellular calcium levels of HUVECs after treatment (*n* = 4). (**F**) Representative plots and quantification showing intracellular ROS levels of HUVECs after treatment (*n* = 4). (**G**) Representative plots and quantification showing TMRM levels of HUVECs after treatment (*n* = 4). (**H**) Representative plots and quantification showing TUNEL levels of HUVECs after treatment (*n* = 4). (**I**) Expression levels of p38, ERK1/2, caspase-3 and Bcl-2 from HUVECs with/without hypoxic treatment. (**J**) Schematic illustration showing that HTR-8/SVneo cells were cultured under LPS conditions, and the collected supernatant was co-cultured with neutrophils and HUVECs under SX682 treatment. (**K**) Confocal microscopy images showing NETs formation of neutrophils with different treatment. Scale bar: 200 μm. (**L**) Fluorescence images in live/dead staining experiments of HUVECs. Scale bar: 200 μm. (M) Representative flow cytometry plots and quantification of cell apoptosis of HUVECs (*n* = 4). (**N**) Representative plots and quantification showing intracellular calcium levels of HUVECs after treatment (*n* = 4). (**O**) Representative plots and quantification showing intracellular ROS levels of HUVECs after treatment (*n* = 4). (**P**) Representative plots and quantification showing TMRM levels of HUVECs after treatment (*n* = 4). (**Q**) Representative plots and quantification showing TUNEL levels of HUVECs after treatment (*n* = 4). (**R**) Expression and phosphorylation levels of p38, ERK1/2, caspase-3 and Bcl-2 from HUVECs with/without LPS treatment. **p < 0.01, ****p < 0.0001. Statistical values were calculated using one-way ANOVA (D-H, M-Q)

Building on the finding that trophoblast-derived IL-8/CXCL1 drives neutrophil NETosis and endothelial injury, we next asked whether pharmacological interruption of IL-8 signaling at its receptors would blunt this cascade. We used SX682, a small-molecule antagonist of CXCR1/2, in neutrophil-endothelial co-cultures exposed to conditioned media from HTR-8/SVneo cells. Under hypoxic conditioning, neutrophils exposed to hypoxia-derived supernatant formed abundant NETs by confocal microscopy, whereas NET burden was markedly reduced in the presence of SX682 (Fig. 4A-B). Concordantly, HUVECs co-cultured with these neutrophils exhibited extensive cell death by live/dead imaging, which was mitigated by CXCR1/2 inhibition (Fig. 4C). Flow cytometry confirmed that apoptotic HUVECs increased after exposure to hypoxia-conditioned neutrophils and fell significantly with SX682 (Fig. 4D). At the level of organellar stress, SX682 lowered the hypoxia-induced surges in intracellular Ca²⁺ and ROS, restored mitochondrial membrane potential, and reduced nuclear DNA fragmentation (Fig. 4E-H). In line with these functional readouts, trophoblast-primed neutrophils drove a pro-apoptotic signaling shift in HUVECs—reduced p-p38 and p-ERK1/2, diminished Bcl-2 and increased cleaved caspase-3—that was reversed toward baseline by SX682 (Fig. 4I). Together, these data indicate that blockade of neutrophil CXCR1/2 interrupts NET-mediated mitochondrial dysfunction and apoptosis in endothelial cells under hypoxic trophoblast cues.

We next tested whether this protection generalizes to an inflammatory trophoblast program. When neutrophils and HUVECs were exposed to supernatant from LPS-stimulated HTR-8/SVneo cells, neutrophils again underwent robust NETosis that was curtailed by SX682 (Fig. 4J-K). Endothelial damage mirrored the hypoxia setting: live/dead imaging revealed pronounced loss of HUVEC viability after LPS-conditioned neutrophil exposure, with preservation of viable cells when CXCR1/2 was inhibited (Fig. 4L). Annexin V/PI staining showed a significant reduction in apoptotic HUVECs with SX682 (Fig. 4M). SX682 also dampened the LPS-driven elevations in endothelial Ca²⁺ and ROS, rescued mitochondrial membrane potential, and lowered TUNEL positivity (Fig. 4N-Q). Consistently, SX682 normalized stress- and apoptosis-associated proteins: p-p38 and p-ERK1/2 increased toward control levels, Bcl-2 rose, and cleaved caspase-3 declined (Fig. 4R). These parallel results under hypoxic and LPS stimuli argue that CXCR1/2 is a convergent upstream node linking pathologic trophoblast signals to neutrophil activation and secondary endothelial injury.

To evaluate species conservation and the functional relevance of the murine CXCL1 orthologue, we repeated the experiments using primary mouse trophoblasts, bone marrow-derived neutrophils and mouse vascular endothelial cells (MUVECs). In the hypoxia model, SX682 suppressed trophoblast-induced NET formation and protected MUVEC viability, reduced Annexin V/PI apoptosis, lowered Ca²⁺ and ROS, restored mitochondrial membrane potential, and decreased TUNEL signal (Supplementary Figure S7A-S7H). Immunoblotting again showed reversal of the pro-apoptotic signature—normalization of p-p38 and p-ERK1/2, increased Bcl-2 and reduced cleaved caspase-3—in SX682-treated conditions (Supplementary Figure S7I). An analogous protection was observed when mouse trophoblasts were stimulated with LPS, SX682 limited NETosis, preserved endothelial viability, reduced apoptosis and oxidative/ionic stress, and corrected mitochondrial signaling and apoptotic markers (Supplementary Figure S8A-S8I). The concordance between human and mouse systems, and across distinct trophoblast stressors, underscores the robustness of the CXCL1/IL-8–CXCR1/2 axis in orchestrating neutrophil-dependent endothelial damage.

Collectively, these experiments establish that CXCR1/2 inhibition with SX682 is sufficient to disrupt the trophoblast-neutrophil-endothelium inflammatory circuit delineated above. By preventing IL-8/CXCL1-driven NETosis, SX682 attenuates endothelial Ca²⁺ influx and ROS generation, preserves mitochondrial potential, and rebalances p38/ERK and caspase-Bcl-2 signaling toward cell survival. Given that hypoxia and LPS represent clinically relevant triggers in preeclampsia, these data nominate the IL-8–CXCR1/2 axis as a tractable therapeutic target to protect the maternal vasculature from neutrophil-mediated mitochondrial injury.

### CXCL1 neutralization or CXCR1/2 blockade mitigates preeclampsia-like pathology and restores fetal growth in LPS-challenged mice

**Fig. 5:**
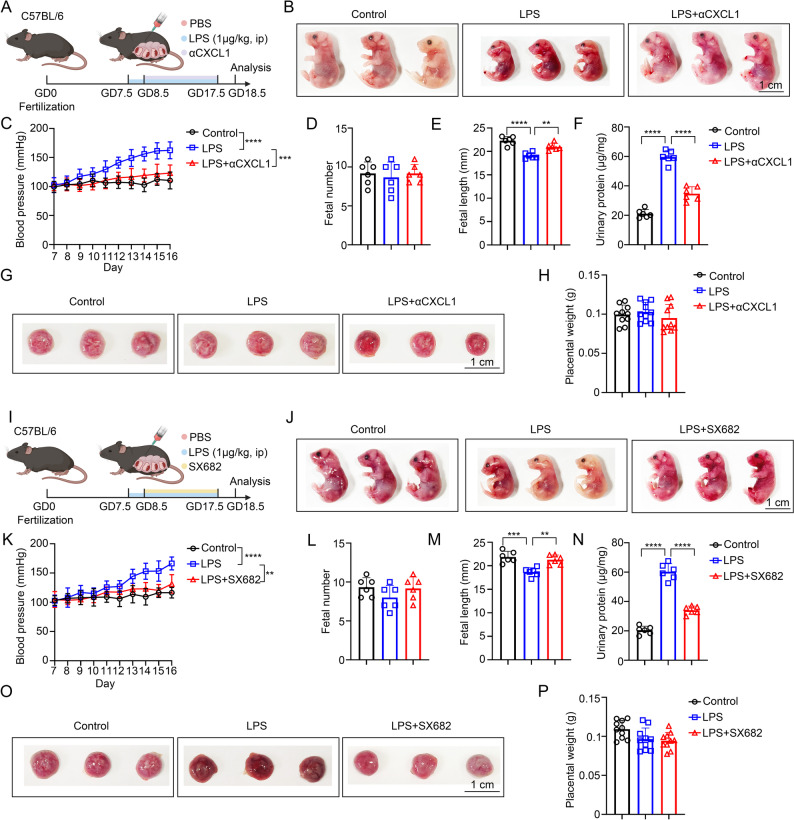
Blockade of CXCL1 or CXCR1/2 signaling alleviates preeclampsia-like symptoms and improves fetal and placental outcomes in LPS-induced mouse models. (**A**) Schematic of in vivo experiment of C57BL/6 mice treated with LPS and αCXCL1. (**B**) Representative images of fetal mice treated with LPS and αCXCL1 (*n* = 3 dams per group). Scale bar, 1 cm. (**C**) Statistical analysis of blood pressure (*n* = 3 dams per group). (**D**) Statistical analysis of fetal number (*n* = 6 litters per group). (**E**) Statistical analysis of fetal length (*n* = 6 litters per group). (**F**) Statistical analysis of urinary protein concentration in pregnant mice (*n* = 6 litters per group). (**G**) Representative images of placentas from mice treated with LPS and αCXCL1 (*n* = 3 dams per group). Scale bar, 1 cm. (**H**) Statistical analysis of placental weight (*n* = 10). (**I**) Schematic of in vivo experiment of C57BL/6 mice treated with LPS and SX682. (**J**) Representative images of fetal mice treated with LPS and SX682 (*n* = 3 dams per group). Scale bar, 1 cm. (**K**) Statistical analysis of blood pressure (*n* = 3 dams per group). (**L**) Statistical analysis of fetal number (*n* = 6 litters per group). (**M**) Statistical analysis of fetal length (*n* = 6 litters per group). (**N**) Statistical analysis of urinary protein concentration in pregnant mice (*n* = 6 litters per group). (**O**) Representative images of placentas from mice treated with LPS and SX682 (*n* = 3 dams per group). Scale bar, 1 cm. (**P**) Statistical analysis of placental weight (*n* = 10). **p < 0.01, ***p < 0.001, ****p < 0.0001. Statistical values were calculated using one-way ANOVA (**E**, **F**, **M**, **N**) and two-way ANOVA (**C**, **K**)

To determine whether interrupting the placental chemokine axis that we identified in vitro can mitigate disease in vivo, we pharmacologically targeted the mouse IL-8 ortholog CXCL1 and its cognate receptors CXCR1/2 in a gestational LPS model of preeclampsia-like pathology. Pregnant C57BL/6 females received LPS at GD7.5 to induce maternal hypertension, proteinuria and fetal growth restriction, followed by intervention at GD8.5 either with a neutralizing anti-CXCL1 antibody (αCXCL1) or the small-molecule CXCR1/2 antagonist SX682, and were analysed at GD18.5 (Fig. 5).

Neutralization of CXCL1 improved both maternal and fetal readouts. Gross inspection of fetuses showed that LPS exposure shortened fetal length, an effect visibly corrected by αCXCL1 (Fig. 5A-B). Longitudinal tail-cuff measurements demonstrated that αCXCL1 blunted the progressive rise in maternal blood pressure observed after LPS challenge (Fig. 5C). Restoration of fetal growth was confirmed quantitatively: fetal length, reduced by LPS, increased toward control values with αCXCL1 (Fig. 5D-E). Consistent with these benefits, urinary protein concentrations were significantly reduced by αCXCL1 (Fig. 5F). By contrast, placental weight was not altered across groups, and placental gross morphology remained indistinguishable, indicating that chemokine blockade chiefly relieved functional vascular injury rather than producing overt macroscopic changes in placental mass (Fig. 5G-H).

Blocking chemokine signaling at the receptor level yielded convergent results. Dams treated with SX682 after LPS exhibited fetuses with visibly larger size relative to the LPS group, and maternal blood pressure trajectories were significantly attenuated (Fig. 5I-K). Proteinuria likewise decreased with SX682, and fetal length improved toward control (Fig. 5L-N). As with αCXCL1, SX682 did not measurably affect litter size or placental weight and did not change gross placental appearance, again pointing to preservation of vascular function rather than changes in placental bulk (Fig. 5O-P).

To further evaluate the clinical translatability of targeting the IL-8/CXCL1-CXCR1/2 axis, we tested whether intervention initiated after the onset of PE-like symptoms would still confer therapeutic benefit. Instead of initiating treatment at GD8.5 (as in Fig. 5), we delayed administration of either neutralizing anti-CXCL1 antibody or the CXCR1/2 antagonist SX682 until GD12.5, a time point after the establishment of measurable hypertension and proteinuria. As shown in Supplementary Figure S9A-S9H, delayed intervention with αCXCL1 significantly attenuated maternal hypertension, reduced proteinuria, and improved fetal length compared to LPS-treated controls. Similarly, delayed administration of SX682 produced significant therapeutic benefits, including reduced blood pressure, enhanced fetal growth, and decreased proteinuria (Supplementary Figure S9I-S9P). However, the magnitude of these improvements was notably reduced compared with early intervention initiated at GD8.5. These findings demonstrate that while IL-8/CXCL1-CXCR1/2 blockade remains therapeutically beneficial even after disease onset, which supporting clinical translatability that earlier intervention yields greater efficacy, highlighting the importance of timely diagnosis and treatment in clinical PE management.

Together, these findings validate the IL-8/CXCL1-CXCR1/2 pathway as a tractable driver of neutrophil-mediated endothelial injury in preeclampsia-like disease and support its therapeutic targeting. As summarized in the graphical abstract, stressed trophoblasts release IL-8 (CXCL1 in mice) that recruits and activates neutrophils to form NETs within the placental milieu; NETs-associated cytotoxicity compromises endothelial survival, increases vascular permeability and propagates maternal hypertension and proteinuria. Interrupting this chemokine circuit at either the ligand or receptor level breaks the feed-forward loop, protecting endothelial cells and improving maternal and fetal outcomes. These data situate neutrophil chemokine signaling upstream of placental vascular dysfunction and nominate CXCL1-CXCR1/2 blockade as a promising strategy to ameliorate preeclampsia features in vivo (Fig. 6).

## Discussion

Preeclampsia remains a leading cause of maternal and perinatal morbidity and mortality worldwide, characterized by hypertension, proteinuria, and placental dysfunction [1]. Although the etiology of PE is multifactorial, maternal endothelial injury is widely recognized as a central pathological feature that contributes to systemic vascular dysfunction and adverse pregnancy outcomes [3]. In the present study, we identify IL-8 as a key trophoblast-derived chemokine that links placental stress to neutrophil activation and NET formation. By integrating patient samples, in vitro co-culture systems, and complementary murine models, we demonstrate that trophoblast-derived IL-8 promotes neutrophil NETosis, which in turn induces endothelial mitochondrial dysfunction and apoptosis. These findings define a trophoblast-neutrophil-endothelium inflammatory axis that amplifies vascular injury in PE. Importantly, pharmacological interruption of this pathway through CXCL1 neutralization or CXCR1/2 inhibition alleviated hypertension, proteinuria, and fetal growth restriction in vivo, highlighting IL-8-CXCR1/2 signaling as a potential therapeutic target.

Our results place the IL-8-driven NETosis pathway within the broader pathogenic framework of PE. Placental hypoxia and inflammatory stress are well-established drivers of trophoblast dysfunction and contribute to the release of anti-angiogenic mediators such as soluble fms-like tyrosine kinase-1 (sFlt-1), a key factor implicated in endothelial injury in PE [6–7]. In this context, trophoblast-derived IL-8 may represent an upstream inflammatory signal linking placental stress to neutrophil recruitment and NET formation. NET-associated oxidative stress, mitochondrial dysfunction, and inflammatory mediator release may further exacerbate endothelial injury and interact with established mechanisms such as angiogenic imbalance and oxidative stress that characterize PE [10]. These findings suggest that IL-8-mediated NETosis represents an inflammatory component that integrates immune activation with vascular dysfunction in the maternal circulation.

Although NET formation has been reported in several inflammatory conditions, including infection-associated pregnancy complications and systemic inflammatory diseases [27]. Our data suggest that this pathway may be particularly amplified in PE. Persistent placental hypoxia and dysregulated trophoblast signaling may sustain IL-8 production and create a feed-forward inflammatory loop that promotes continuous neutrophil activation at the maternal-fetal interface. In this setting, excessive NET formation may contribute to endothelial dysfunction and vascular inflammation, thereby exacerbating maternal hypertension and organ injury [17]. Thus, while NETosis is not unique to PE, the trophoblast-derived IL-8 axis may represent a disease-relevant mechanism linking placental stress to systemic vascular pathology.

Several limitations of the experimental models used in this study should also be considered. We employed both LPS-induced inflammatory PE models and adenovirus-mediated sFlt-1 overexpression models to reproduce key clinical features of PE. These models capture distinct aspects of disease pathology: the LPS model primarily reflects the inflammatory and immune-driven components of PE, whereas the sFlt-1 model recapitulates the anti-angiogenic imbalance associated with endothelial dysfunction [15]. However, neither model fully reproduces the chronic and heterogeneous progression of human PE. Therefore, while our findings support a mechanistic role for IL-8-mediated NETosis in endothelial injury, further validation in additional models and human studies will be required to fully define the contribution of this pathway to PE pathogenesis.

From a translational perspective, targeting the IL-8-CXCR1/2 pathway may offer new therapeutic opportunities. CXCR1/2 antagonists such as reparixin and SX682 have been explored in inflammatory diseases and oncology, although their clinical efficacy has sometimes been limited by compensatory chemokine signaling and concerns regarding impaired host defense [28]. Similarly, anti-cytokine therapies targeting inflammatory pathways have shown variable success in vascular diseases due to disease heterogeneity and pathway redundancy [29]. These considerations highlight the importance of carefully evaluating therapeutic context and dosing strategies when translating chemokine-targeted approaches to PE. Combination strategies targeting both inflammatory and angiogenic pathways, as well as biomarker-guided patient stratification based on circulating IL-8 or NET-associated markers, may improve therapeutic efficacy.

Another important consideration is the timing and safety of interventions during pregnancy. In our study, early pharmacological blockade of CXCR1/2 effectively prevented the development of PE-like features, while delayed intervention after the onset of disease manifestations still provided measurable benefit, suggesting that the IL-8-NETosis axis remains therapeutically targetable during disease progression. Nevertheless, systemic inhibition of IL-8 signaling may theoretically influence maternal host defense or fetal immune development, as IL-8 plays a physiological role in neutrophil recruitment. Future therapeutic strategies may therefore benefit from approaches that minimize systemic exposure, such as localized placental delivery or short-term interventions during defined disease windows. Additional studies in larger animal models and human tissues will be required to fully evaluate the long-term safety and translational feasibility of targeting this pathway during pregnancy.

## Conclusions

Collectively, our study establishes IL-8–driven neutrophil NETosis as a central mediator linking trophoblast stress to maternal endothelial injury in PE. By providing direct mechanistic evidence for this trophoblast–neutrophil–endothelium cascade and demonstrating the therapeutic benefit of interrupting IL-8 signaling, we offer a new conceptual framework for understanding PE immunopathogenesis. These findings not only expand fundamental knowledge of maternal–fetal immune interactions but also highlight a novel therapeutic avenue to mitigate maternal vascular dysfunction and improve fetal outcomes. Targeting the IL-8–CXCR1/2 axis may thus represent a promising immunomodulatory strategy for preeclampsia and related pregnancy complications.

## Supplementary Information

Below is the link to the electronic supplementary material.

Supplementary Material 1

Supplementary Material 2

## Data Availability

The datasets used and/or analysed during the current study are available from the corresponding author on reasonable request.

## Abbreviations

GD: Gestational day
HUVECs: Human vascular endothelial cells
LPS: Lipopolysaccharide
MACS: Magnetic-activated cell sorting
MUVECs: Mouse vascular endothelial cells
NETs: Neutrophil extracellular traps
PE: Preeclampsia
PI: Propidium iodide
STR: Short tandem repeat
