# Supplementary material for: IL-8–driven neutrophil NETosis triggers endothelial apoptosis and exacerbates preeclampsia
Source: J Transl Med. 2026 Apr 4;24:501. doi: 10.1186/s12967-026-08084-3 (PMC13069710; doi:10.1186/s12967-026-08084-3)
Supplement: Supplementary file 1 — Supplementary Material 1 [file 12967_2026_8084_MOESM1_ESM.docx]

*Supplementary Material for*

**IL-8–Driven Neutrophil NETosis Triggers Endothelial Apoptosis and Exacerbates Preeclampsia**

Wushan Li^1,3,#,*^, Xinyuan Li^2,#^, Xuemei Liu^2^, Mingjie Zhang^3^, Wei Li^1^, Xinlin Jiao^1^, Fengchun Gao^3, *^, Baoxia Cui^1, *^

^1^ Department of Obstetrics and Gynecology, Qilu Hospital of Shandong University, Cheeloo College of Medicine, Shandong University, 107 Cultural West Road, Jinan, Shandong Province, 250012, China

^2^ Department of Immunology, Shandong Provincial Key Laboratory of Infection & Immunology, School of Basic Medical Sciences, Cheeloo College of Medicine, Shandong University, Jinan, Shandong Province 250012, China.

^3^ Department of Obstetrics, Jinan Maternity and Child Care Hospital Affiliated to Shandong First Medical University, Jinan, Shandong Province, 250000, China

^#^ These authors contributed equally to this work.

^*^ Corresponding author: Baoxia Cui, E-mail: cuibaoxia@sdu.edu.cn; Fengchun Gao: fengchungaoyx@sina.cn; Wushan Li: [liwushan2005@163.com](mailto:liwushan2005@163.com).


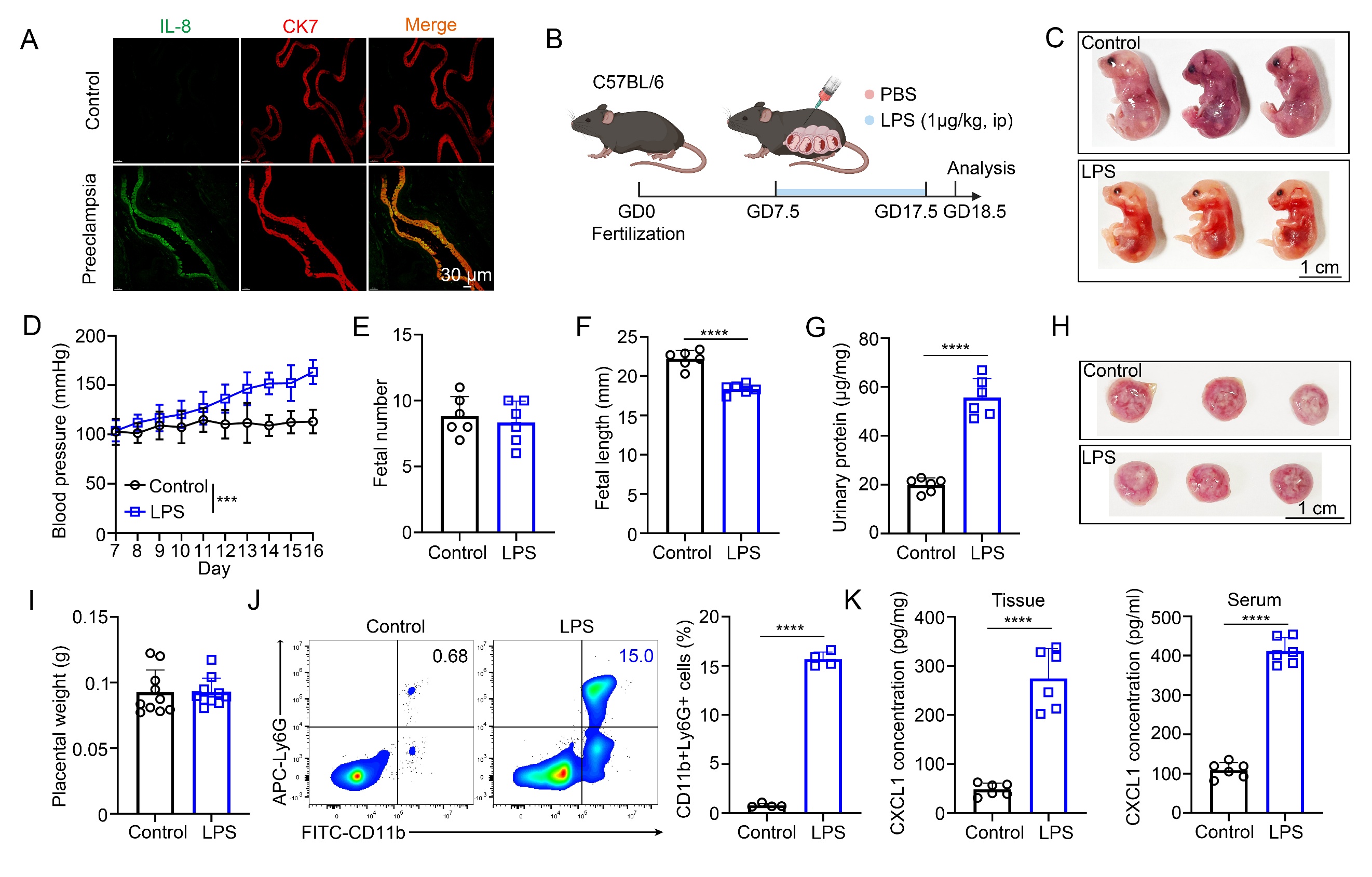


**Supplementary Figure S1. LPS-induced mouse model recapitulates preeclampsia-like features and CXCL1 upregulation.** (A) Representative immunofluorescence images of placental sections from healthy controls (upper panels) and patients with preeclampsia (lower panels). Sections were stained with anti-IL-8 antibody (green), and anti-CK7 antibody (red). Scale bar, 30 μm. (B) Schematic of *in vivo* experiment of C57BL/6 mice treated with LPS. (C) Representative images of fetal mice treated with LPS (n=3). Scale bar, 1 cm. (D) Statistical analysis of blood pressure (n=3). (E) Statistical analysis of fetal number (n=6). (F) Statistical analysis of fetal length (n=6). (G) Statistical analysis of urinary protein concentration in pregnant mice (n=6). (H) Representative images of placenta from mice treated with LPS (n=3). Scale bar, 1 cm. (I) Statistical analysis of placental weight (n=10). (J) Gating strategy, representative plots and quantification diagrams of mouse CD11b^+^Ly6G^+^ neutrophils in placental tissue from mice treated with LPS. (K) CXCL1 concentration in tissue and serum from mice treated with LPS (n=6). ****p<0.0001. Statistical values were calculated using one-way ANOVA (F, G, J, K) and two-way ANOVA (D).


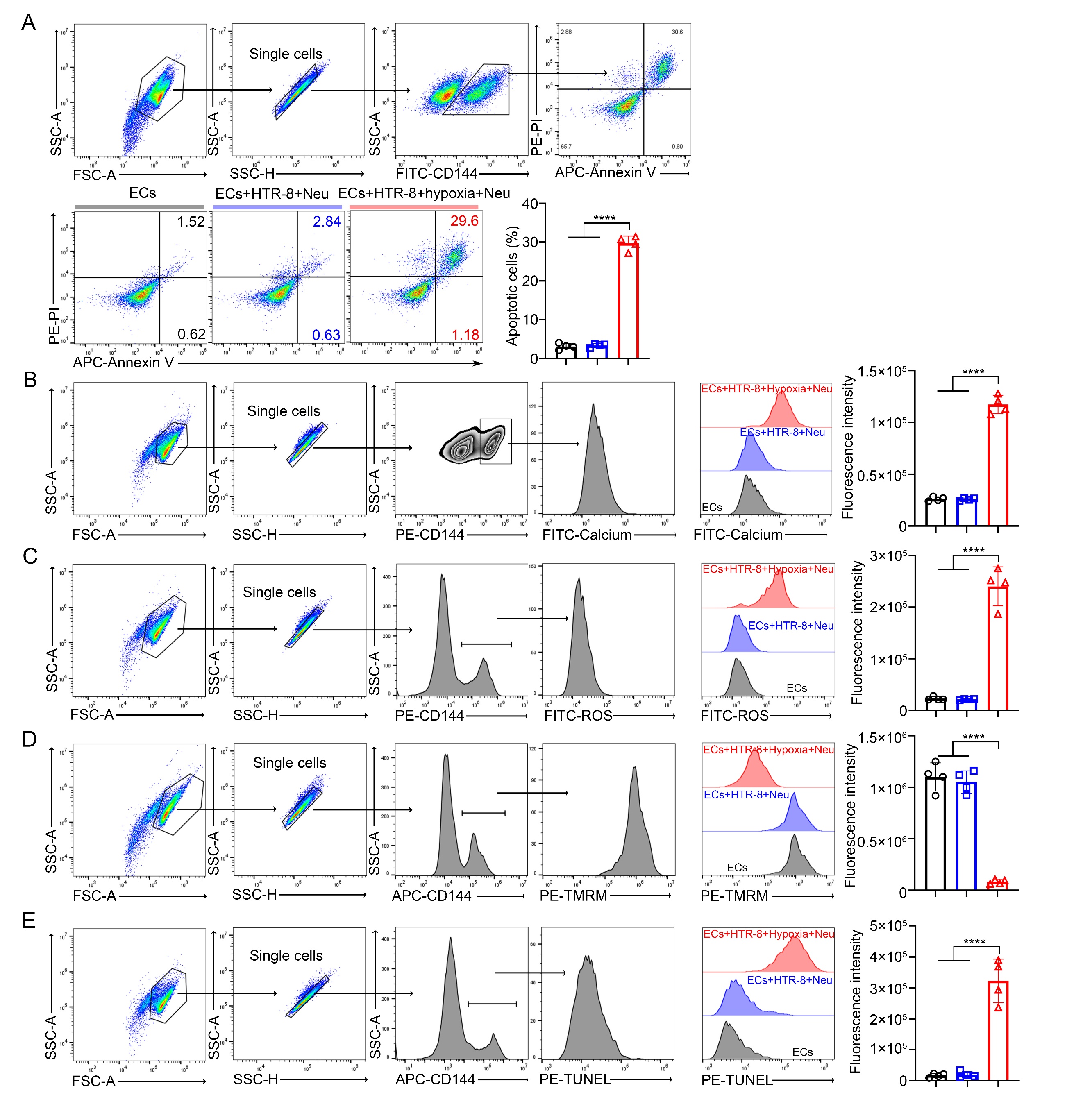


**Supplementary Figure S2. Hypoxia-stimulated trophoblasts promote IL-8–dependent neutrophil NETosis that triggers endothelial apoptosis and dysfunction.** (A) Gating strategy, representative flow cytometry plots and quantification of cell apoptosis of HUVECs (n=4). (B) Gating strategy, representative plots and quantification diagrams showing intracellular calcium level of HUVECs after treatment (n=4). (C) Gating strategy, representative plots and quantification diagrams showing intracellular ROS level of HUVECs after treatment (n=4). (D) Gating strategy, representative plots and quantification diagrams showing TMRM level of HUVECs after treatment (n=4). (E) Gating strategy, representative plots and quantification diagrams showing TUNEL level of HUVECs after treatment (n=4). ****p<0.0001. Statistical values were calculated using one-way ANOVA (A-E).


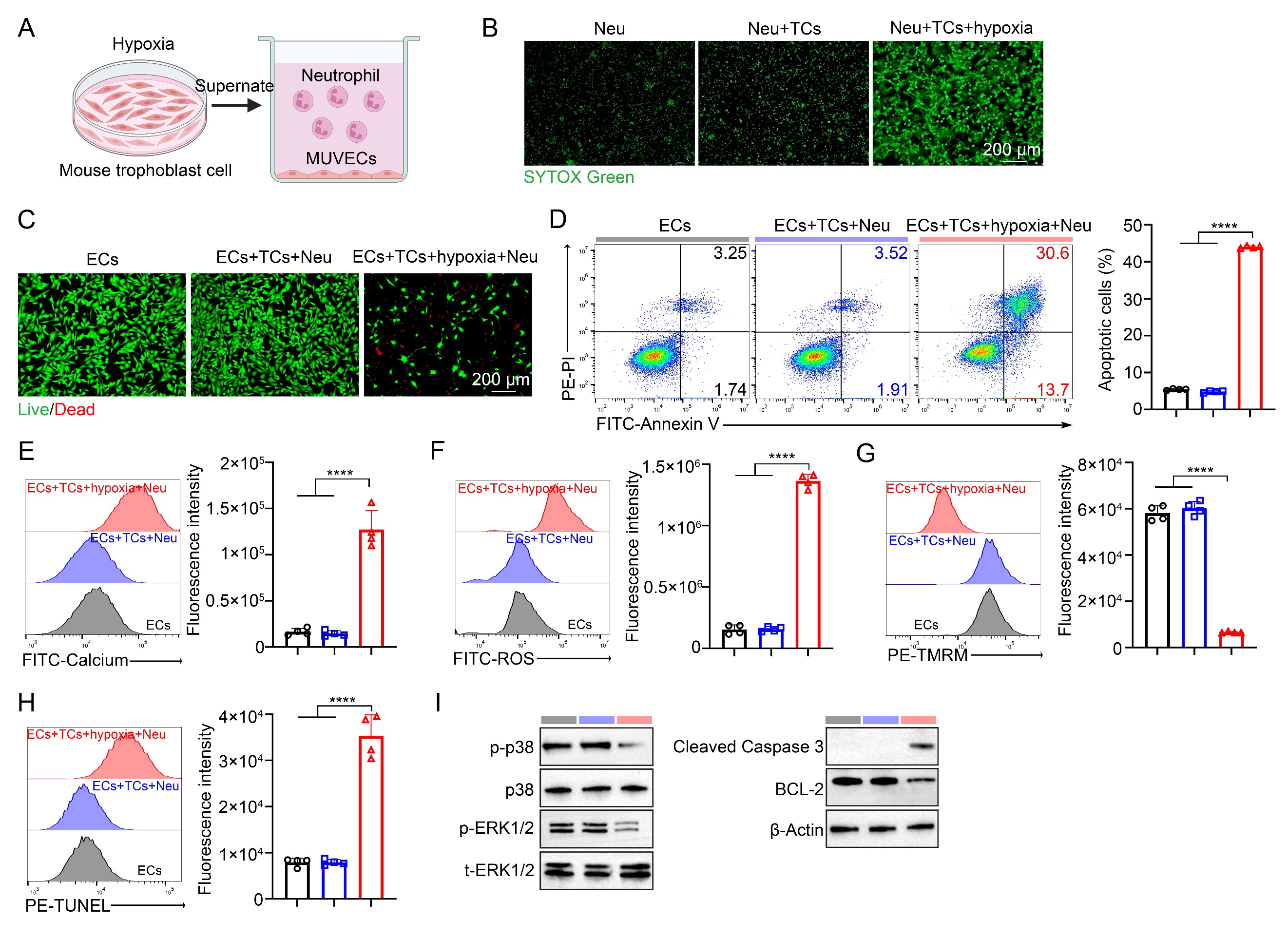


**Supplementary Figure S3. Hypoxic mouse trophoblasts induce neutrophil NETosis and endothelial cell apoptosis.** (A) Schematic illustration showing that mouse trophoblast cells were cultured under hypoxic conditions, and the collected supernatant was co-cultured with neutrophils and MUVECs. (B) Confocal microscopy images showing NETs formation of neutrophils with different treatment. Scale bar, 200 μm. (C) Fluorescence images in live/dead staining experiments of MUVECs. Scale bar, 200 μm. (D) Representative flow cytometry plots and quantification of cell apoptosis of MUVECs (n=4). (E) Representative plots and quantification diagrams showing intracellular calcium level of MUVECs after treatment (n=4). (F) Representative plots and quantification diagrams showing intracellular ROS level of MUVECs after treatment (n=4). (G) Representative plots and quantification diagrams showing TMRM level of MUVECs after treatment (n=4). (H) Representative plots and quantification diagrams showing TUNEL level of MUVECs after treatment (n=4). (I) The expression and phosphorylation levels of p38, ERK1/2, caspase-3 and Bcl-2 from MUVECs with/without hypoxic treatment. ****p<0.0001. Statistical values were calculated using one-way ANOVA (D-H).


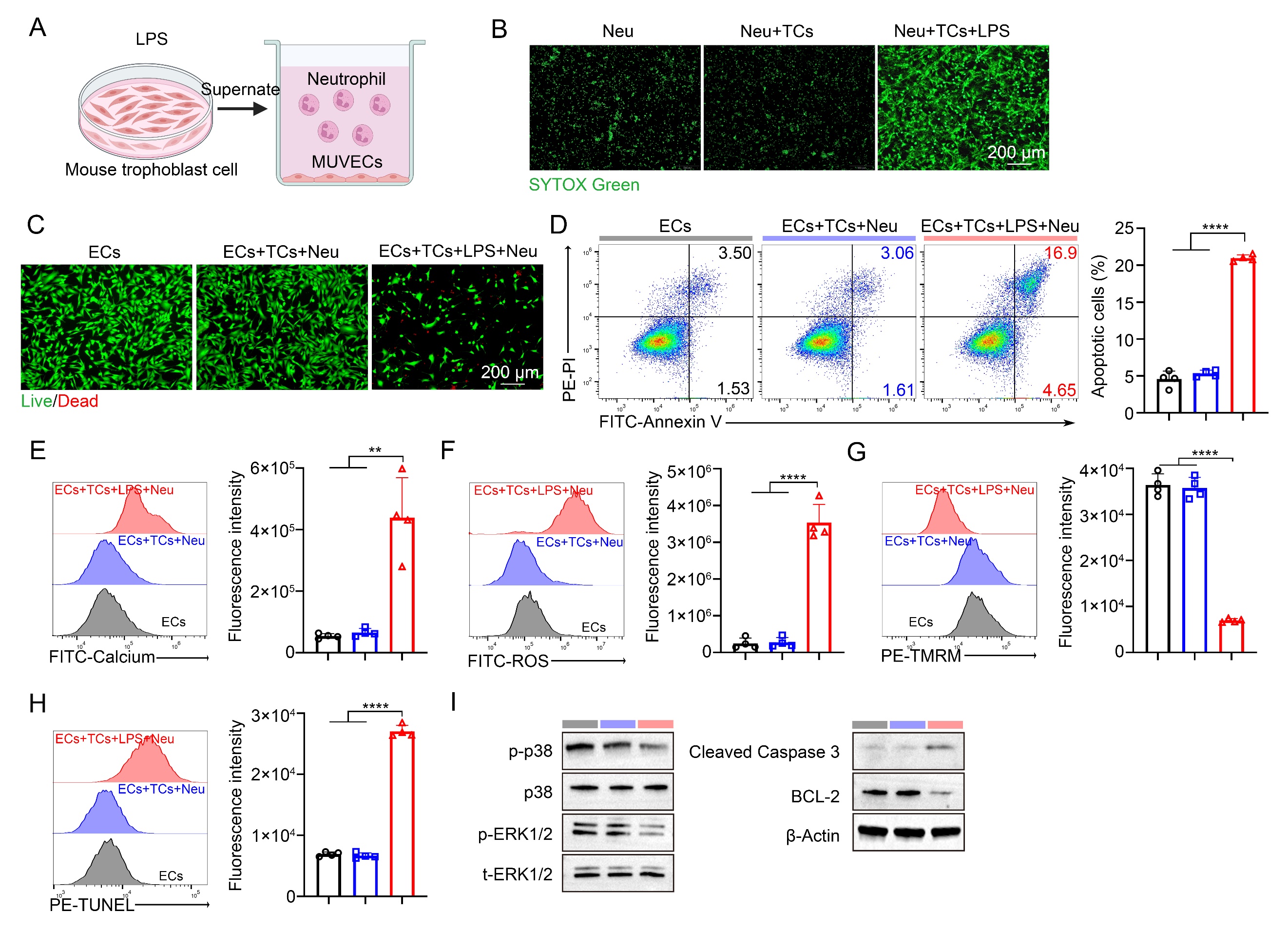


**Supplementary Figure S4. LPS-stimulated trophoblasts promote neutrophil NETosis and endothelial cell injury.** (A) Schematic illustration showing that mouse trophoblast cells were cultured under LPS conditions, and the collected supernatant was co-cultured with neutrophils and MUVECs. (B) Confocal microscopy images showing NETs formation of neutrophils with different treatment. Scale bar, 200 μm. (C) Fluorescence images in live/dead staining experiments of MUVECs. Scale bar, 200 μm. (D) Representative flow cytometry plots and quantification of cell apoptosis of MUVECs (n=4). (E) Representative plots and quantification diagrams showing intracellular calcium level of MUVECs after treatment (n=4). (F) Representative plots and quantification diagrams showing intracellular ROS level of MUVECs after treatment (n=4). (G) Representative plots and quantification diagrams showing TMRM level of MUVECs after treatment (n=4). (H) Representative plots and quantification diagrams showing TUNEL level of MUVECs after treatment (n=4). (I) The expression and phosphorylation levels of p38, ERK1/2, caspase-3 and Bcl-2 from HUVECs with/without LPS treatment. **p<0.01, ****p<0.0001. Statistical values were calculated using one-way ANOVA (D-H).


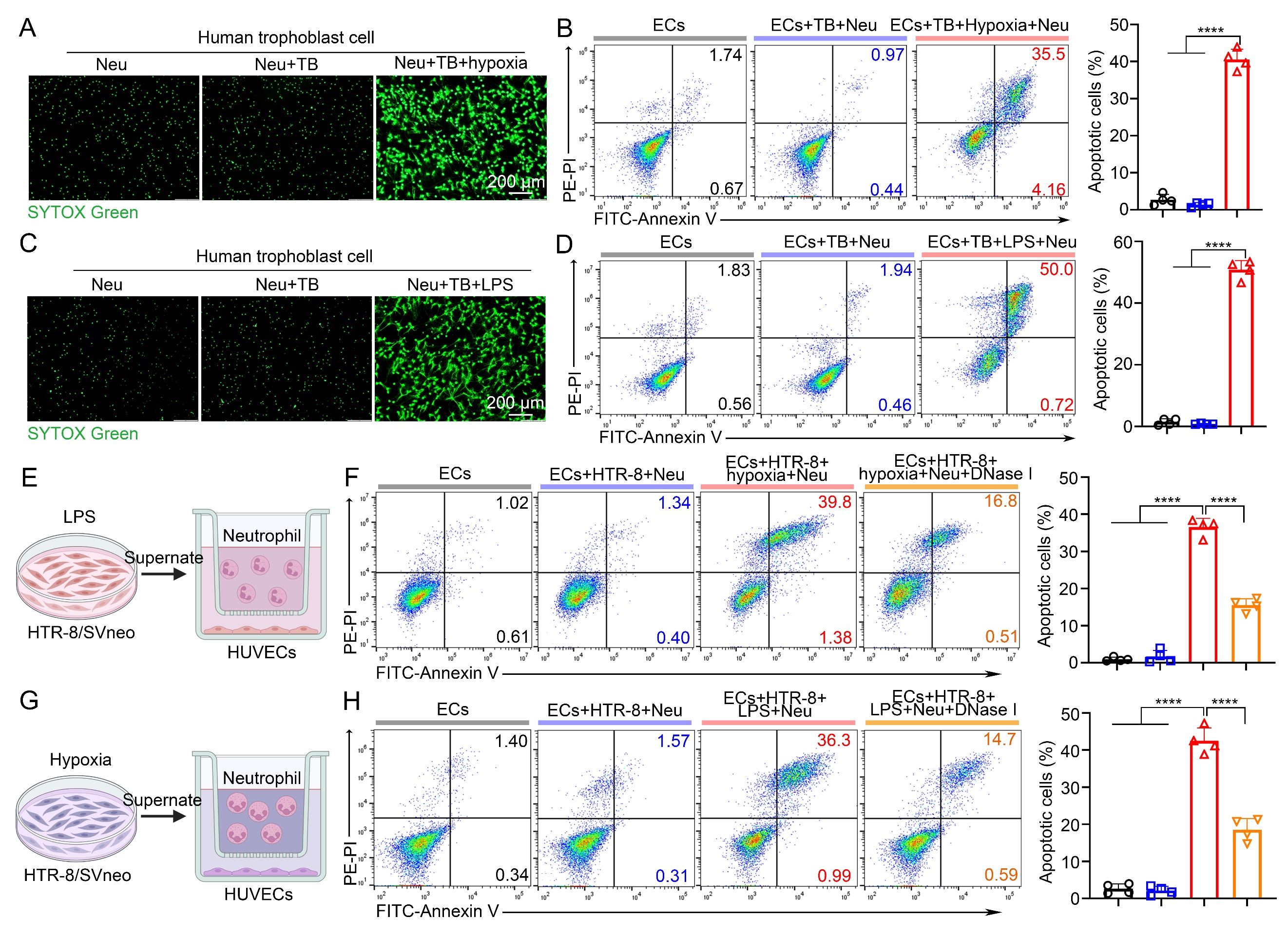


**Supplementary Figure S5. Validation of trophoblast-derived IL-8/CXCL1 and NETs-mediated endothelial injury in human and mouse systems** (A) Confocal microscopy images showing NETs formation of neutrophils with different treatment. Scale bar, 200 μm. (B) Representative flow cytometry plots and quantification of cell apoptosis of HUVECs (n=4). (C) Confocal microscopy images showing NETs formation of neutrophils with different treatment. Scale bar, 200 μm. (D) Representative flow cytometry plots and quantification of cell apoptosis of HUVECs (n=4). (E) Schematic illustration showing that HTR-8/SVneo cells were cultured under LPS conditions, and the collected supernatant was co-cultured with neutrophils and HUVECs using transwell plates. (F) Representative flow cytometry plots and quantification of cell apoptosis of HUVECs (n=4). (G) Schematic illustration showing that HTR-8/SVneo cells were cultured under hypoxic conditions, and the collected supernatant was co-cultured with neutrophils and HUVECs using transwell plates. (H) Representative flow cytometry plots and quantification of cell apoptosis of HUVECs (n=4). ****p<0.0001. Statistical values were calculated using one-way ANOVA (B, D, F, H).


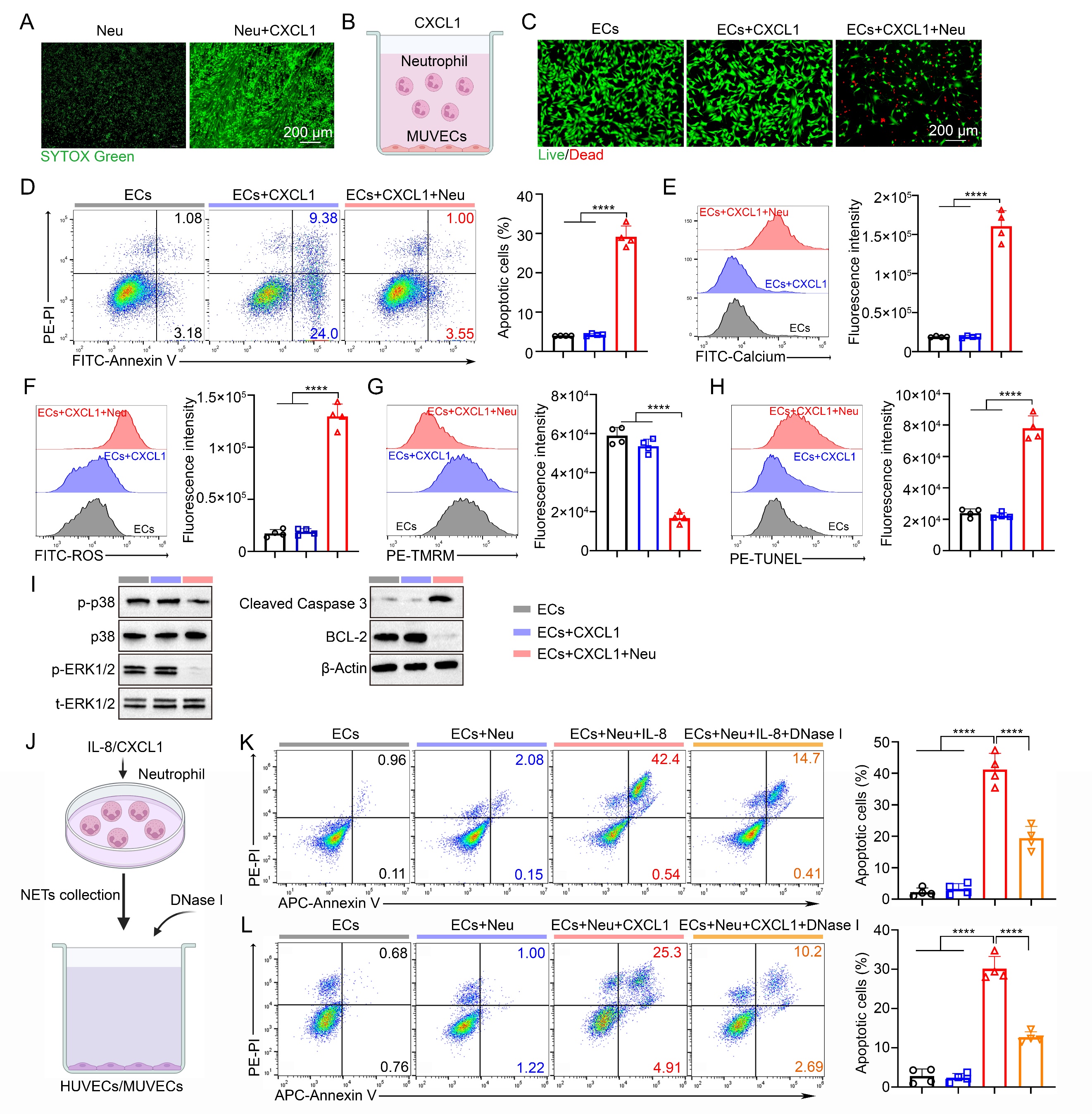


**Supplementary Figure S6. IL-8 promotes neutrophil NETosis and endothelial cell apoptosis.** (A) Confocal microscopy images showing NETs formation of neutrophils with/without IL-8 treatment. Scale bar, 200 μm. (B) Schematic illustration showing that MUVECs were co-cultured with neutrophils. (C) Fluorescence images in live/dead staining experiments of MUVECs. Scale bar, 200 μm. (D) Representative flow cytometry plots and quantification of cell apoptosis of MUVECs (n=4). (E) Representative plots and quantification diagrams showing intracellular calcium level of MUVECs after treatment (n=4). (F) Representative plots and quantification diagrams showing intracellular ROS level of MUVECs after treatment (n=4). (G) Representative plots and quantification diagrams showing TMRM level of MUVECs after treatment (n=4). (H) Representative plots and quantification diagrams showing TUNEL level of MUVECs after treatment (n=4). (I) The expression and phosphorylation levels of p38, ERK1/2, caspase-3 and Bcl-2 from MUVECs with different treatment. (J) Schematic illustration showing that human/mouse derived neutrophils were treated with IL-8/CXCL1, and the collected NETs were co-cultured with HUVECs/MUVECs with/without DNase I treatment. (K) Representative flow cytometry plots and quantification of cell apoptosis of HUVECs (n=4). (L) Representative flow cytometry plots and quantification of cell apoptosis of MUVECs (n=4). ****p<0.0001. Statistical values were calculated using one-way ANOVA (D-H, K-L).


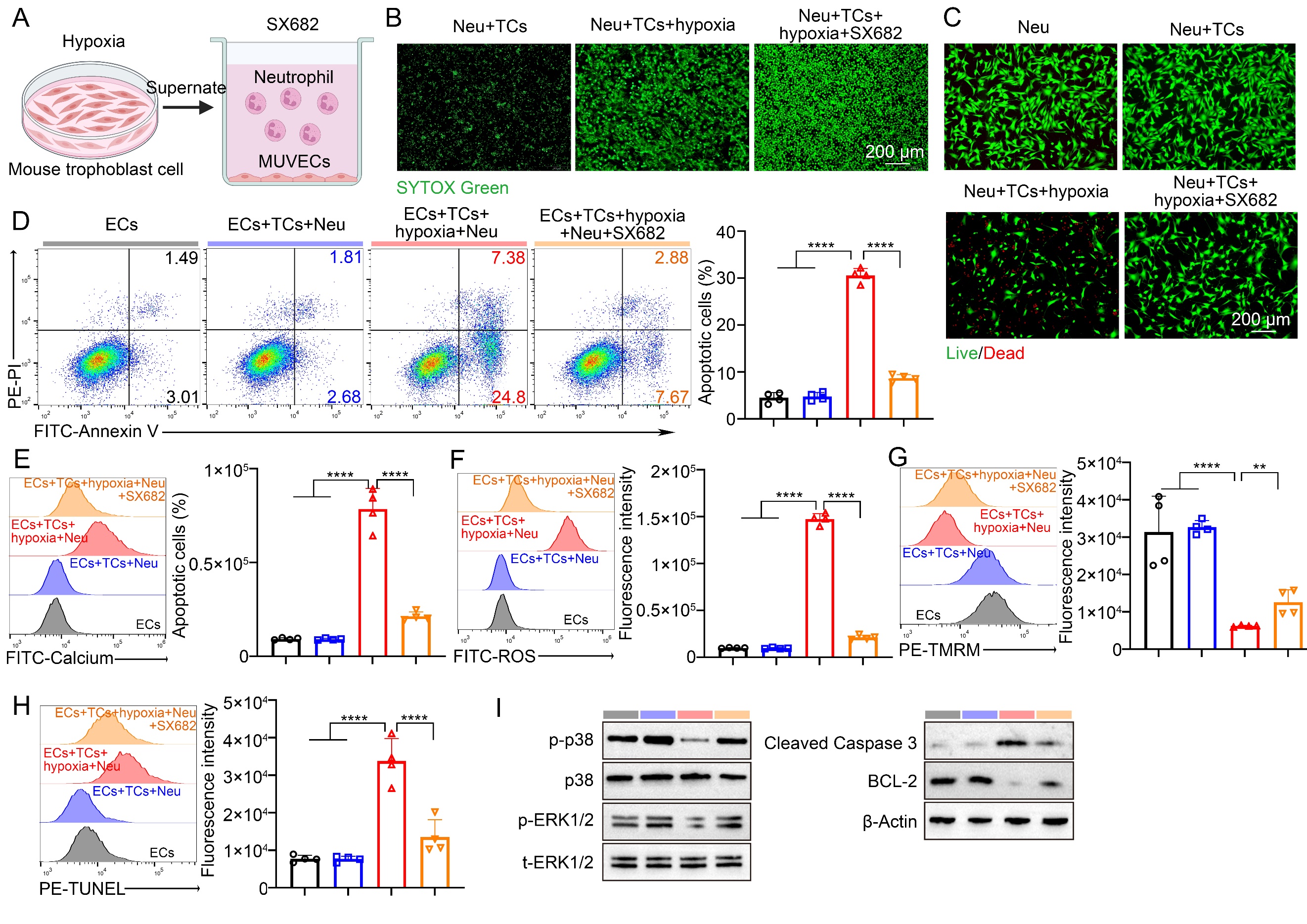


**Supplementary Figure S7. SX682 attenuates hypoxia-induced neutrophil NETosis and endothelial apoptosis.** (A) Schematic illustration showing that mouse trophoblast cells were cultured under hypoxic conditions, and the collected supernatant was co-cultured with neutrophils and MUVECs under SX682 treatment. (B) Confocal microscopy images showing NETs formation of neutrophils with different treatment. Scale bar, 200 μm. (C) Fluorescence images in live/dead staining experiments of MUVECs. Scale bar, 200 μm. (D) Representative flow cytometry plots and quantification of cell apoptosis of MUVECs (n=4). (E) Representative plots and quantification diagrams showing intracellular calcium level of MUVECs after treatment (n=4). (F) Representative plots and quantification diagrams showing intracellular ROS level of MUVECs after treatment (n=4). (G) Representative plots and quantification diagrams showing TMRM level of MUVECs after treatment (n=4). (H) Representative plots and quantification diagrams showing TUNEL level of MUVECs after treatment (n=4). (I) The expression and phosphorylation levels of p38, ERK1/2, caspase-3 and Bcl-2 from MUVECs with/without hypoxic treatment. **p<0.01, ****p<0.0001. Statistical values were calculated using one-way ANOVA (D-H).


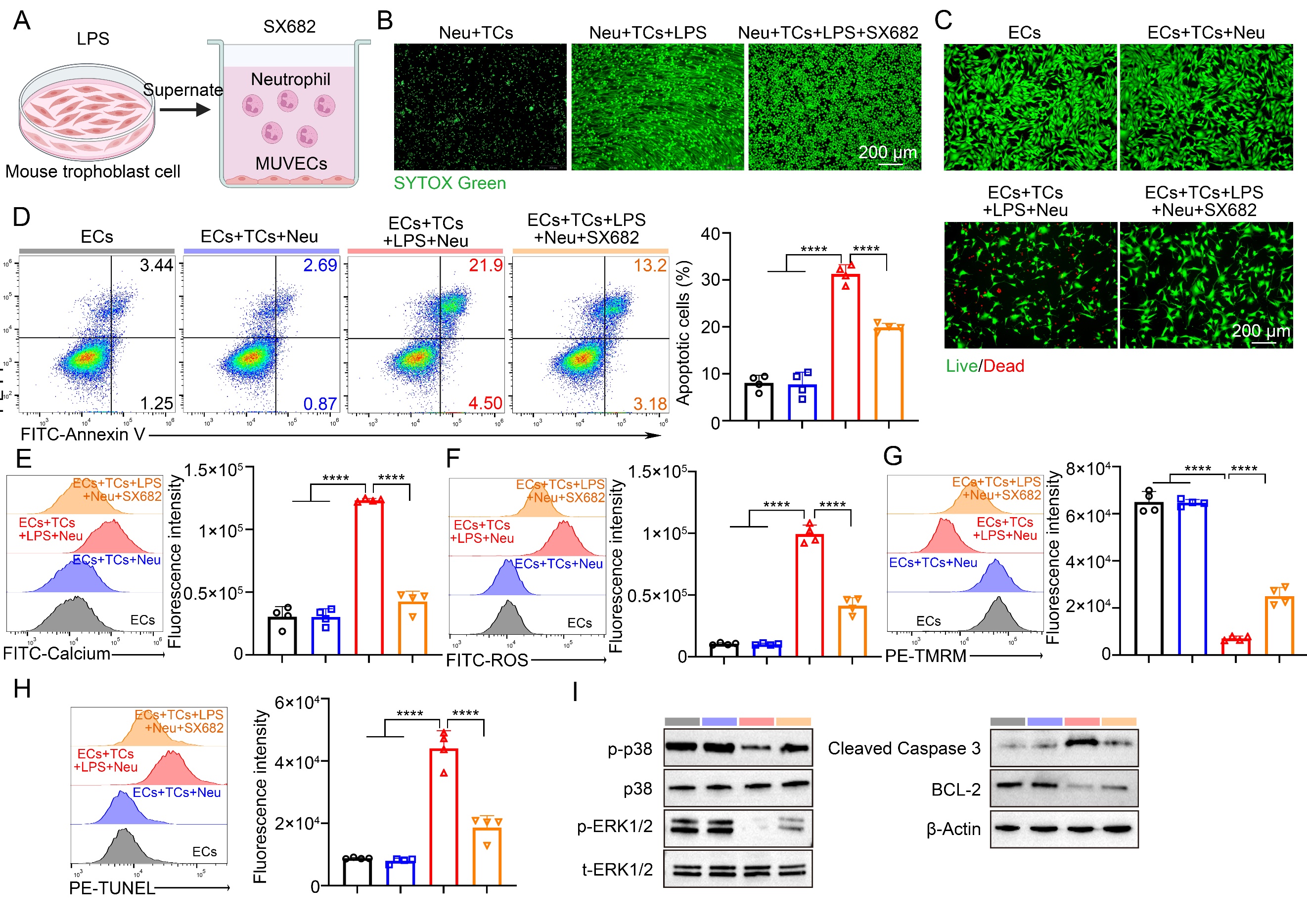


**Supplementary Figure S8. SX682 alleviates LPS-induced neutrophil NETosis and endothelial apoptosis.** (A) Schematic illustration showing that mouse trophoblast cells were cultured under LPS conditions, and the collected supernatant was co-cultured with neutrophils and MUVECs under SX682 treatment. (B) Confocal microscopy images showing NETs formation of neutrophils with different treatment. Scale bar, 200 μm. (C) Fluorescence images in live/dead staining experiments of MUVECs. Scale bar, 200 μm. (D) Representative flow cytometry plots and quantification of cell apoptosis of MUVECs (n=4). (E) Representative plots and quantification diagrams showing intracellular calcium level of MUVECs after treatment (n=4). (F) Representative plots and quantification diagrams showing intracellular ROS level of MUVECs after treatment (n=4). (G) Representative plots and quantification diagrams showing TMRM level of MUVECs after treatment (n=4). (H) Representative plots and quantification diagrams showing TUNEL level of MUVECs after treatment (n=4). (I) The expression and phosphorylation levels of p38, ERK1/2, caspase-3 and Bcl-2 from HUVECs with/without LPS treatment. ****p<0.0001. Statistical values were calculated using one-way ANOVA (D-H).


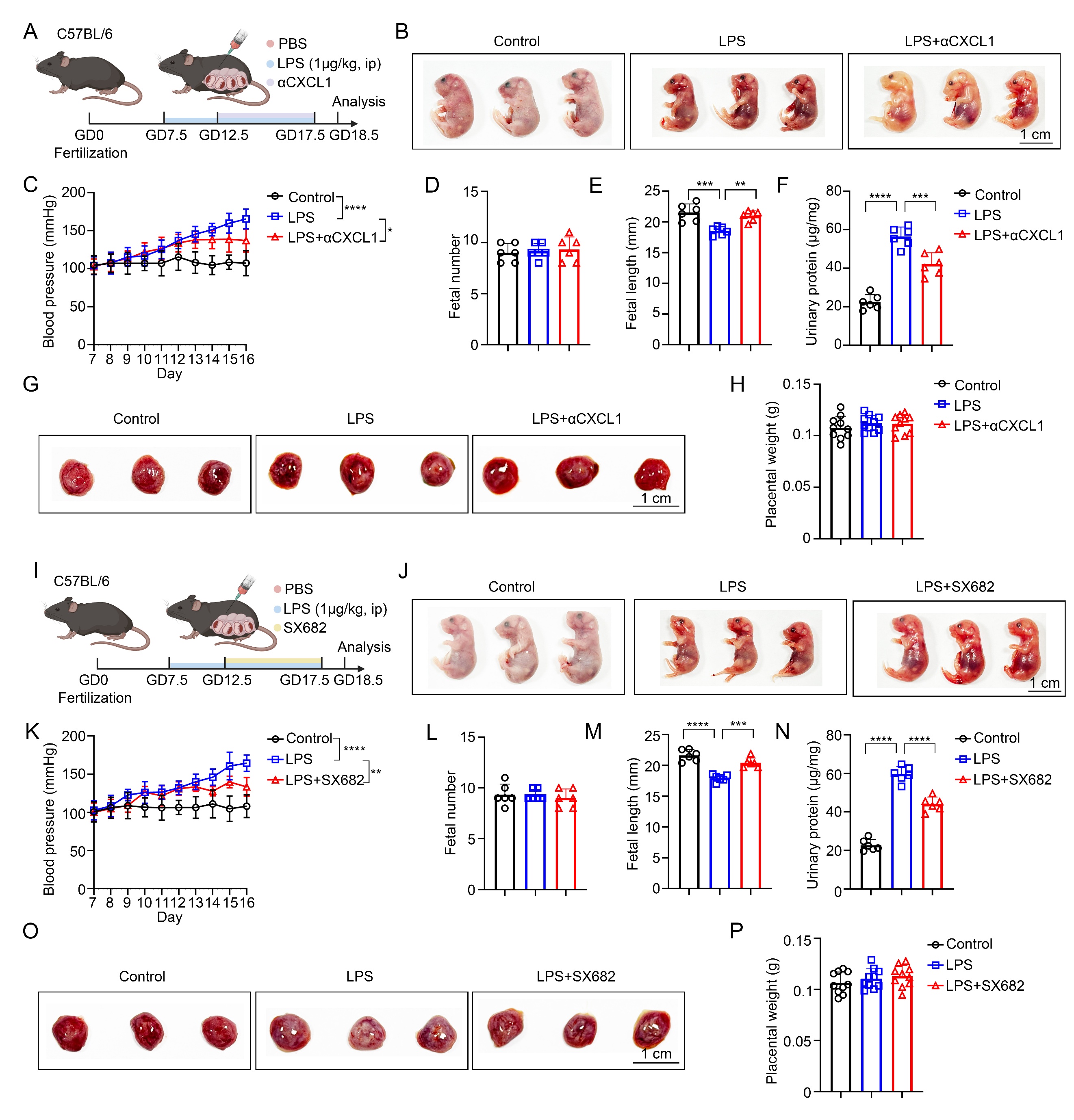


**Supplementary Figure S9. Delayed intervention with CXCL1-CXCR1/2 blockade partially rescues PE.** (A) Schematic of *in vivo* experiment of C57BL/6 mice treated with LPS and αCXCL1. (B) Representative images of fetal mice treated with LPS and αCXCL1 (n=3). Scale bar, 1 cm. (C) Statistical analysis of blood pressure (n=3). (D) Statistical analysis of fetal number (n=6). (E) Statistical analysis of fetal length (n=6). (F) Statistical analysis of urinary protein concentration in pregnant mice (n=6). (G) Representative images of placenta from mice treated with LPS and αCXCL1 (n=3). Scale bar, 1 cm. (H) Statistical analysis of placental weight (n=10). (I) Schematic of *in vivo* experiment of C57BL/6 mice treated with LPS and SX682. (J) Representative images of fetal mice treated with LPS and SX682 (n=3). Scale bar, 1 cm. (K) Statistical analysis of blood pressure (n=3). (L) Statistical analysis of fetal number (n=6). (M) Statistical analysis of fetal length (n=6). (N) Statistical analysis of urinary protein concentration in pregnant mice (n=6). (O) Representative images of placenta from mice treated with LPS and SX682 (n=3). Scale bar, 1 cm. (P) Statistical analysis of placental weight (n=10). *p<0.05, **p<0.01, ***p<0.001, ****p<0.0001. Statistical values were calculated using one-way ANOVA (E, F, M, N) and two-way ANOVA (C, K).

**Supplementary Table 1. Clinical Characteristics of the Study Population by Preeclampsia Severity**

| **Characteristics** | **Control**  **(n = 10)** | **Mild PE**  **(n = 10)** | **Severe PE**  **(n = 10)** | **P value** |
| --- | --- | --- | --- | --- |
| Maternal Age (years) | 29.0 ± 3.2 | 29.5 ± 4.1 | 27.6 ± 4.1 | 0.487 |
| Pre-pregnancy BMI (kg/m²) | 22.3 ± 6.0 | 26.5 ± 5.2 | 24.9 ± 4.5 | 0.215 |
| Primiparity, n (%) | 5 (50) | 6 (60) | 7 (70) | 0.671 |
| Gestational Age at Delivery (weeks) | 36.2 ± 1.2 | 37.1 ± 1.0 | 33.4 ± 2.0* | <0.001 |
| Systolic BP (mmHg) | 121.0 ± 6.7 | 138.2 ± 8.1* | 160.0 ± 14.2* | <0.001 |
| Diastolic BP (mmHg) | 75.1 ± 7.8 | 92.4 ± 3.6* | 100.5 ± 11.0* | <0.001 |
| Proteinuria (by dipstick), n (%) |  |  |  | <0.001 |
| Negative | 10 (100) | 3 (30) | 0 (0) |  |
| Trace / 1+ | 0 (0) | 6 (60) | 1 (10) |  |
| ≥ 2+ | 0 (0) | 1 (10) | 9 (90) |  |
| Fetal Birth Weight (g) | 2635 ± 321 | 2526 ± 367 | 1848 ± 505* | <0.001 |
| Fetal Sex (Male), n (%) | 6 (60) | 5 (50) | 6 (60) | 0.873 |
| Apgar score at 1 min | 9.8 ± 0.4 | 9.7 ± 0.5 | 9.0 ± 0.0* | <0.001 |
| Delivery by Cesarean, n (%) | 6 (60) | 9 (90) | 10 (100) | 0.025 |
