# Supplementary figures and images for: IL-8–driven neutrophil NETosis triggers endothelial apoptosis and exacerbates preeclampsia
Source: J Transl Med. 2026 Apr 4;24:501. doi: 10.1186/s12967-026-08084-3 (PMC13069710; doi:10.1186/s12967-026-08084-3)

Figure 2I

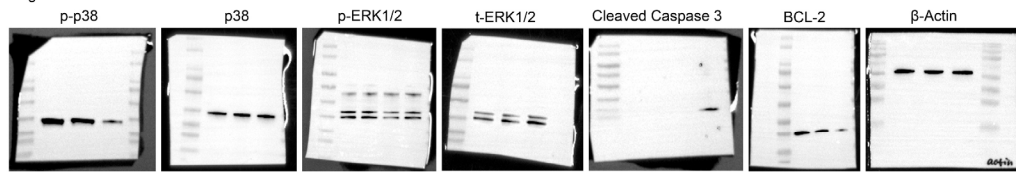

Figure 2R

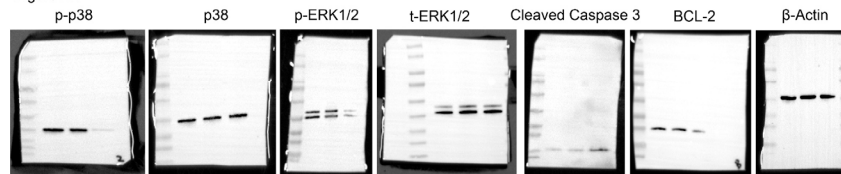

Figure 3I

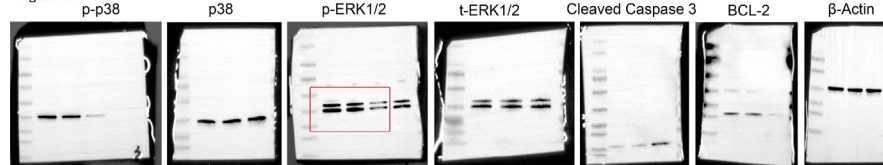

Figure 4I

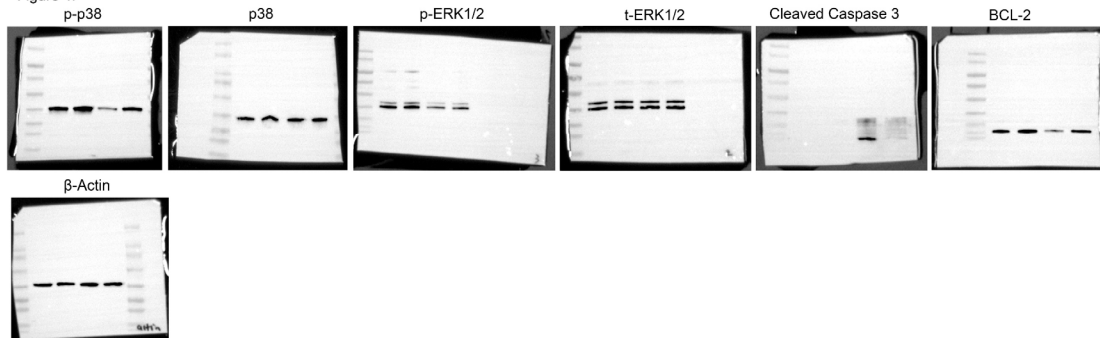

Figure 4R

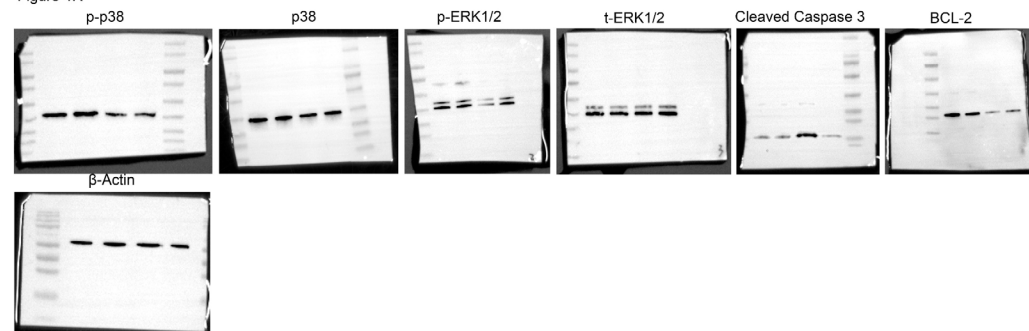

Figure S2l

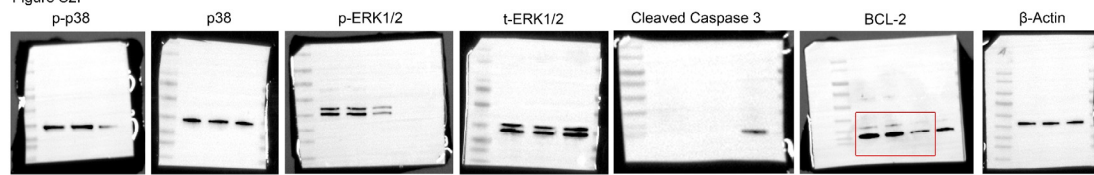

Figure S3l

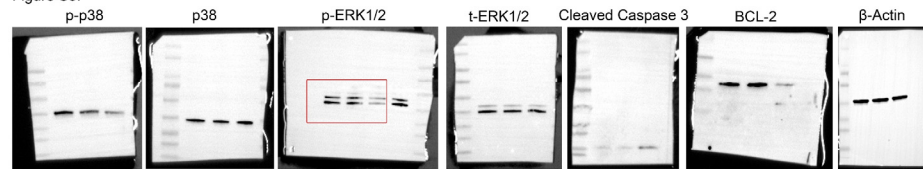

Figure S4l

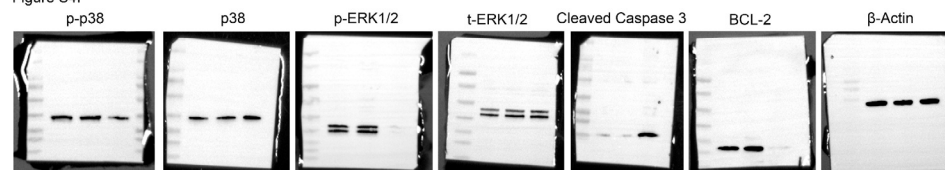

Figure S5l

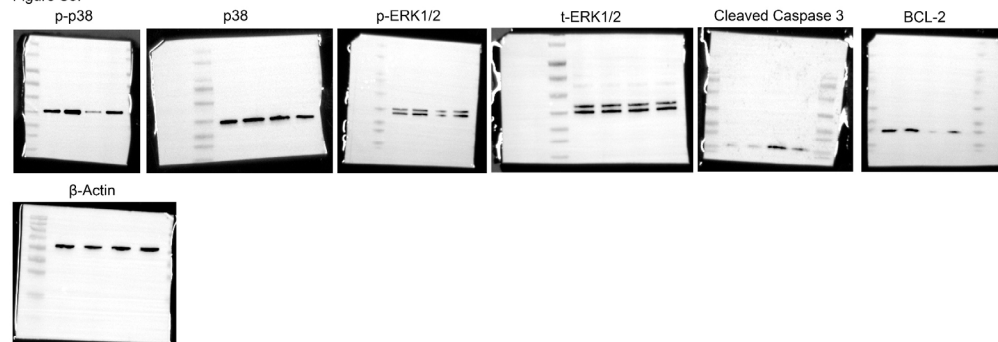

Figure S6l

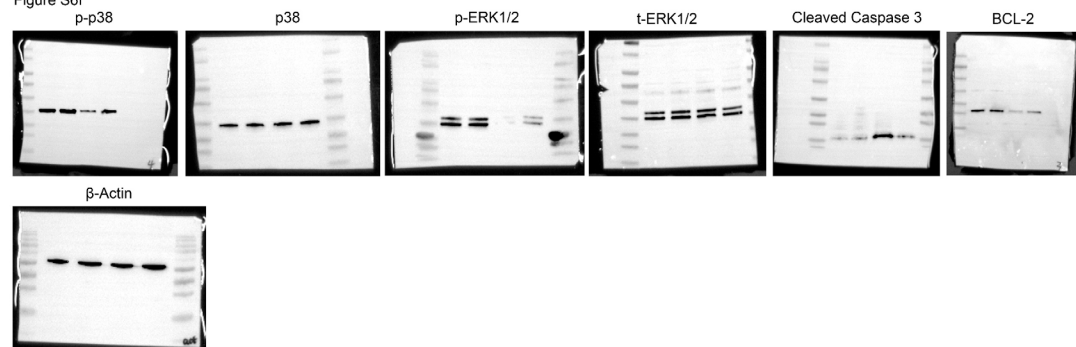

Supplement: Supplementary file 2 — Supplementary Material 2 [file 12967_2026_8084_MOESM2_ESM.pdf]
